# Ferroptosis mediated by the IDO1/Kyn/AhR pathway triggers acute thymic involution in sepsis

**DOI:** 10.1038/s41419-025-07882-9

**Published:** 2025-07-25

**Authors:** Zimei Cheng, Kexin Wang, Yixue Wang, Tingyan Liu, Jingjing Li, Yaodong Wang, Weiming Chen, Reyihangu Awuti, Hetian Zhou, Wenjia Tong, Zhenhao Yu, Yao Wang, Guoyun Su, Weiguo Yang, Yufeng Zhou, Guoping Lu, Caiyan Zhang

**Affiliations:** 1 Department of Emergency and Critical Care Medicine, Children’s Hospital of Fudan University, Shanghai Institute of Infectious Disease and Biosecurity, and Institutes of Biomedical Sciences Fudan University, 200032 Shanghai, China; 2 National Health Commission Key Laboratory of Neonatal Diseases, Fudan University, Shanghai, China; 3 Department of Pediatric Critical Care Unit, Anhui Provincial Children’s Hospital, Hefei, China; 4 Center for Molecular Medicine, Children’s Hospital of Fudan University, National Children’s Medical Center, Shanghai, China; 5 Pediatric Intensive Care Unit, Shenzhen Children’s Hospital, Shenzhen, China; 6 Fujian Key Laboratory of Neonatal Diseases, Xiamen Key Laboratory of Neonatal Diseases, Xiamen Children’s Hospital (Children’s Hospital of Fudan University at Xiamen), Xiamen, China

**Keywords:** Experimental models of disease, Immune cell death, Sepsis, Cell death and immune response

## Abstract

Acute thymic involution (ATI) is frequently observed during sepsis, however the underlying mechanisms remain poorly understood. This study demonstrates that ferroptosis plays a crucial role in sepsis-associated ATI. We found that pediatric sepsis patients showed significantly elevated kynurenine (Kyn)/tryptophan (Trp) ratios, indicating increased indoleamine 2,3-dioxygenase 1 (IDO1) activity, along with higher Kyn levels compared to controls. Moreover, Kyn levels were negatively correlated with thymus-to-thorax ratio. Further mechanistic analysis revealed that the enhanced expression of IDO1, induced by inflammatory signals, drives the accumulation of Kyn and subsequent activation of the aryl hydrocarbon receptor (AhR), triggering lipid oxidation-related gene transcription and ferroptosis in thymocytes during sepsis. Treatment with 1-methyltryptophan (IDO1 inhibitor) effectively restore thymic function and improve survival in septic mice. Our findings reveal a novel role for the IDO1/Kyn/AhR pathway in ferroptosis, suggesting that targeting this pathway may offer a promising therapeutic strategy for sepsis.

**Figure:**
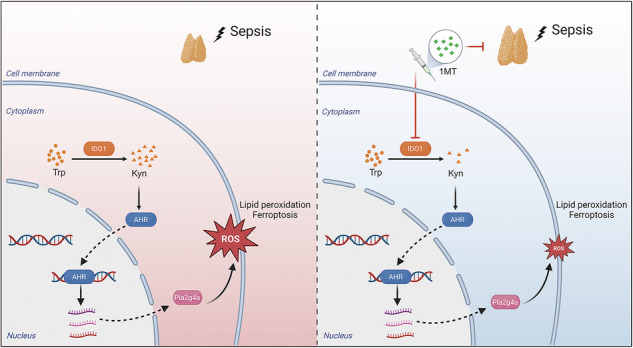
Created with BioRender (https://app.biorender.com/).

Created with BioRender (https://app.biorender.com/).

## Introduction

Sepsis is a life-threatening condition resulting from a dysregulated host response to infection and is characterized by a dual-phase profile of hyperinflammation and immunosuppression, leading to widespread organ injury [1]. Children are particularly vulnerable to sepsis, representing more than half of global cases [2]. The dysregulation of immune cells from both the innate and adaptive immune systems in response to severe infections significantly impairs pathogen clearance and heightens the risk of secondary infections, contributing to the high mortality reported in sepsis [3]. Understanding the mechanisms that disrupt immune homeostasis is, therefore, critical for developing more effective clinical strategies to manage sepsis.

The thymus is pivotal in the maturation, selection, and release of naive T lymphocytes, and supports immunocompetence by producing thymic hormones that enhance lymphopoiesis and T cell function in peripheral lymphoid tissues [4]. Under normal physiological conditions, the thymus develops in concert with other organs and systems, reaching peak size at puberty before gradually undergoing involution. The thymus is crucial for maintaining immune function, not only in childhood but throughout adulthood [5]. Recent evidence indicates that adults who undergo thymectomy are at increased risk of all-cause mortality, cancer, and autoimmune diseases [6]. In children, the thymus is particularly essential for establishing a robust immune defense, as demonstrated by diminished immune responses to vaccines in children post-thymectomy [7]. During the COVID-19 pandemic, children’s more robust immune defense against COVID-19 may be attributed to their relatively intact thymic function, while adults experience age-related thymic atrophy, leading to decreased naive T cell production and weakened immune responses [8]. Acute thymic involution (ATI), accompanied by compromised proliferation, activation, and secretion of T cells, is frequently observed during severe infections [9]. In contrast to the age-related chronic thymic involution, sepsis-associated ATI is marked by a swift diminution in thymic size and weight, disruption of the thymic epithelial structure, and a significant reduction in the populations of various thymocyte subsets. Furthermore, sepsis-associated ATI can lead to reduced TCR repertoire diversity, accelerated aging of naive T cells, and imbalances in T cell subsets, all of which resulting in widespread impairment of antigen response [10]. Despite its clinical significance, the precise mechanism governing ATI during sepsis remains ill-defined.

Ferroptosis, an iron-dependent form of regulated cell death, is characterized by lipid peroxidation, mitochondrial shrinkage, reduced mitochondrial cristae, and increased membrane density [11]. Following the onset of ferroptotic cell death, the cells acquire an immunogenic phenotype, which has the potential to augment inflammatory reactions and thereby precipitate further cellular demise [12]. Increasing evidence suggests that ferroptosis appears to play a vital role in the development of sepsis. Ferroptosis has been implicated in the pathogenesis of sepsis-associated damage in organs such as the lung, heart, kidney, intestinal, and liver [13–17]. Additionally, ferroptosis has been identified in immune cells, such as CD4^+^ T cells, macrophages, and dendrite cells during sepsis [18–20]. However, the role of ferroptosis in ATI during sepsis, remains largely unexplored. Here, we hypothesize that ferroptosis may also play a significant role in the ATI process.

In this study, we provide evidence that ferroptosis is pivotal in sepsis-induced ATI in a polymicrobial sepsis model. Our findings demonstrate that indoleamine 2,3-dioxygenase 1 (IDO1) accelerates thymic involution, leading to T cell exhaustion and dysfunction through the rapid induction of ferroptosis. Mechanistically, IDO1 promotes the accumulation of kynurenine (Kyn) within the thymus, which triggers the activation of the aryl hydrocarbon receptor (AhR). Notably, inhibition of IDO1 successfully restored thymic function and mitigated systemic inflammation during sepsis. These findings elucidate the mechanisms underlying ferroptosis-associated ATI in sepsis and identify the IDO1/Kyn/AhR pathway as a promising therapeutic target for preserving thymic function and improving sepsis outcomes.

## Results

### Acute thymic involution in pediatric sepsis and polymicrobial sepsis mouse model

**Fig. 1:**
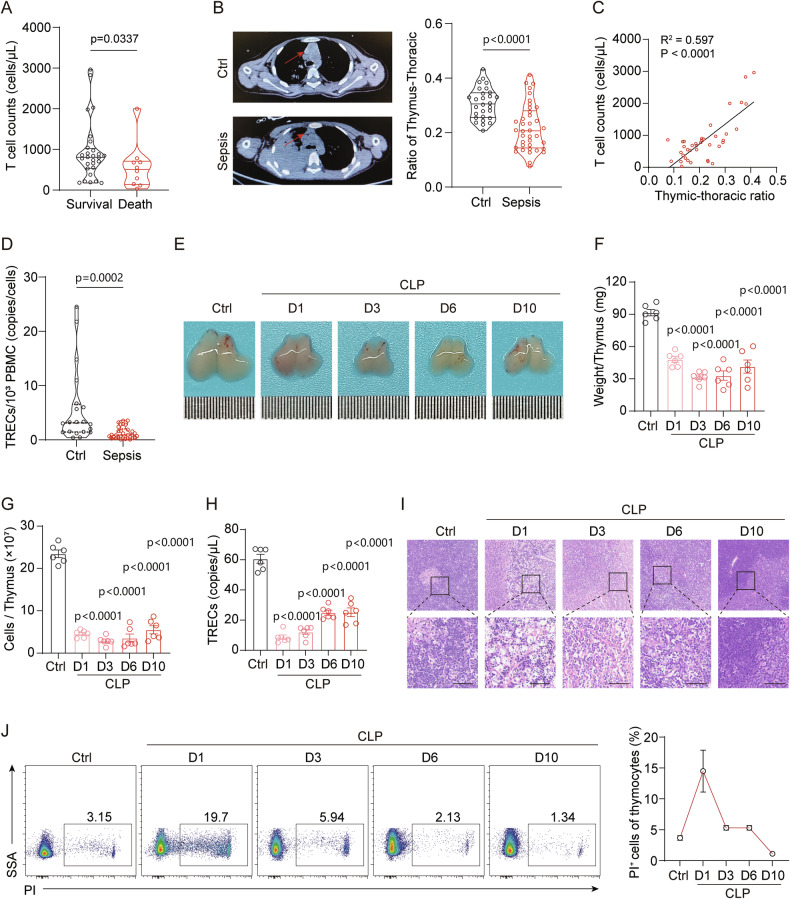
Thymic involution in pediatric sepsis and polymicrobial sepsis mouse model. **A** Peripheral blood T cell counts in surviving (*n* = 28) versus deceased (*n* = 10) pediatric sepsis patients. **B** Representative CT images showing the thymus between sepsis patients (*n* = 38) and controls (Ctrl) (*n* = 28). Violin plots depict the ratio of thymus-to-thoracic cavity in both groups. **C** Correlation between the thymus-to-thoracic ratio and total T cell counts in peripheral blood (*n* = 38). **D** Levels of TRECs in PBMCs from sepsis patients (*n* = 30) and controls (Ctrl) (*n* = 20). **E**−**G** Representative images of thymus appearance (**E**), thymus weight (**F**), and total thymocyte counts (**G**) on days 1, 3, 6, and 10 post-CLP surgery (*n* = 6). **H** Quantification of TRECs in peripheral blood expressed as absolute TRECs counts per microliter of blood (*n* = 6). **I** HE staining of thymus sections on days 1, 3, 6, and 10 post-CLP, showing progressive structural damage (scale bar = 50 μm). **J** Flow cytometry showing PI^+^ (propidium iodide positive) thymocytes at different time points, indicating increased cell death following sepsis (*n* = 4). Bars represent the means ± SEM.

Clinically, decreased T cell counts are significantly associated with poor prognosis in sepsis patients [21]. In our study, we found that T lymphocyte levels were markedly lower in non-survivors compared to survivors (Fig. 1A). T cells are matured and exported as naive T cells from the thymus, which promotes lymphopoiesis and supports T cell function in peripheral lymphoid tissues by secreting thymic hormones [22]. ATI can be triggered by pathogenic infections. Our analysis of thymic tissue imaging from sepsis patients and control children with accident trauma revealed that the thymic-thoracic ratio (TT-ratio) was significantly reduced in septic patients compared to age- and gender-matched control individuals (Fig. 1B). Furthermore, there was a positive correlation between TT-ratio and T cell count, including total T cells, CD4 T cells, and CD8 T cells (Fig. 1C, Fig. S1A, B). T cell receptor excision circles (TRECs) serve as a marker of newly exported thymic cells [23]. To further investigate whether thymic output of T cells is reduced in sepsis, we measured TRECs levels in previously collected pediatric PBMC samples and found that TRECs were significantly reduced in septic children compared to control children (Fig. 1D). Additionally, TT-ratio was negatively correlated with inflammatory markers C-reactive protein (CRP), interleukin-6 (IL-6), and procalcitonin (PCT) (Fig. S1C–E). These results suggest that the relative reduction in thymus size may contribute to the decrease in T cell count in peripheral blood, potentially driven by an excessive early inflammatory response. The clinical information of the enrolled cases is presented in Table S1.

To investigate the mechanisms of sepsis-associated ATI, we established a polymicrobial sepsis model using cecal ligation and puncture (CLP) (Fig. S2). We found that thymus size, weight, and cell count were significantly reduced on the first day after surgery (Fig. 1E–G), reaching their lowest levels on day 3, and these metrics had not returned to normal even by day 10 in surviving mice. The significant decrease in TRECs levels in peripheral blood suggests a diminished capacity of the damaged thymus to export T cells (Fig. 1H). Previous studies suggest that the thymus has a strong capacity for recovery after acute involution [24]. Our study found that in mice surviving the later stages of sepsis, although TRECs levels partially recovered, they remained below healthy levels. The decrease in both the percentage and absolute count of naive CD4^+^ T and naive CD8^+^ T cells further support the above conclusion (Fig. S3A, B). Histological examination revealed disorganized thymic architecture, characterized by indistinct cortical-medullary boundaries and a marked reduction in thymocyte numbers (Fig. 1I). IL-6 and TNF-α levels were elevated in both plasma and thymus tissues at 24 h post-CLP (Fig. S2C–F), which could imply a potential role of inflammatory processes in acute thymic atrophy. These results indicate that sepsis-associated thymic dysfunction persists for an extended period during the recovery phase of sepsis, lasting at least 10 days in the CLP model.

As a key component of the adaptive immune response, the prolonged suppression of thymic immune function is associated with reduced TCR diversity, which can impair the body’s ability to clear primary infections and defend against secondary infections [25]. To elucidate the rationale behind the diminished thymocyte count observed in sepsis, we conducted an investigation into the demise of thymocytes at varying intervals after CLP. It was noted that the percentage of PI^+^ thymocytes of total thymocytes culminated at ~15% at the 24 h mark, subsequently undergoing a progressive recovery (Fig. 1J). Consequently, a thorough examination of the etiology underlying the severe depletion of thymocytes in the initial day after CLP is instrumental in deciphering the mechanisms of ATI mediated by infection.

### Ferroptosis is linked to the death of thymocytes in sepsis-associated ATI

**Fig. 2:**
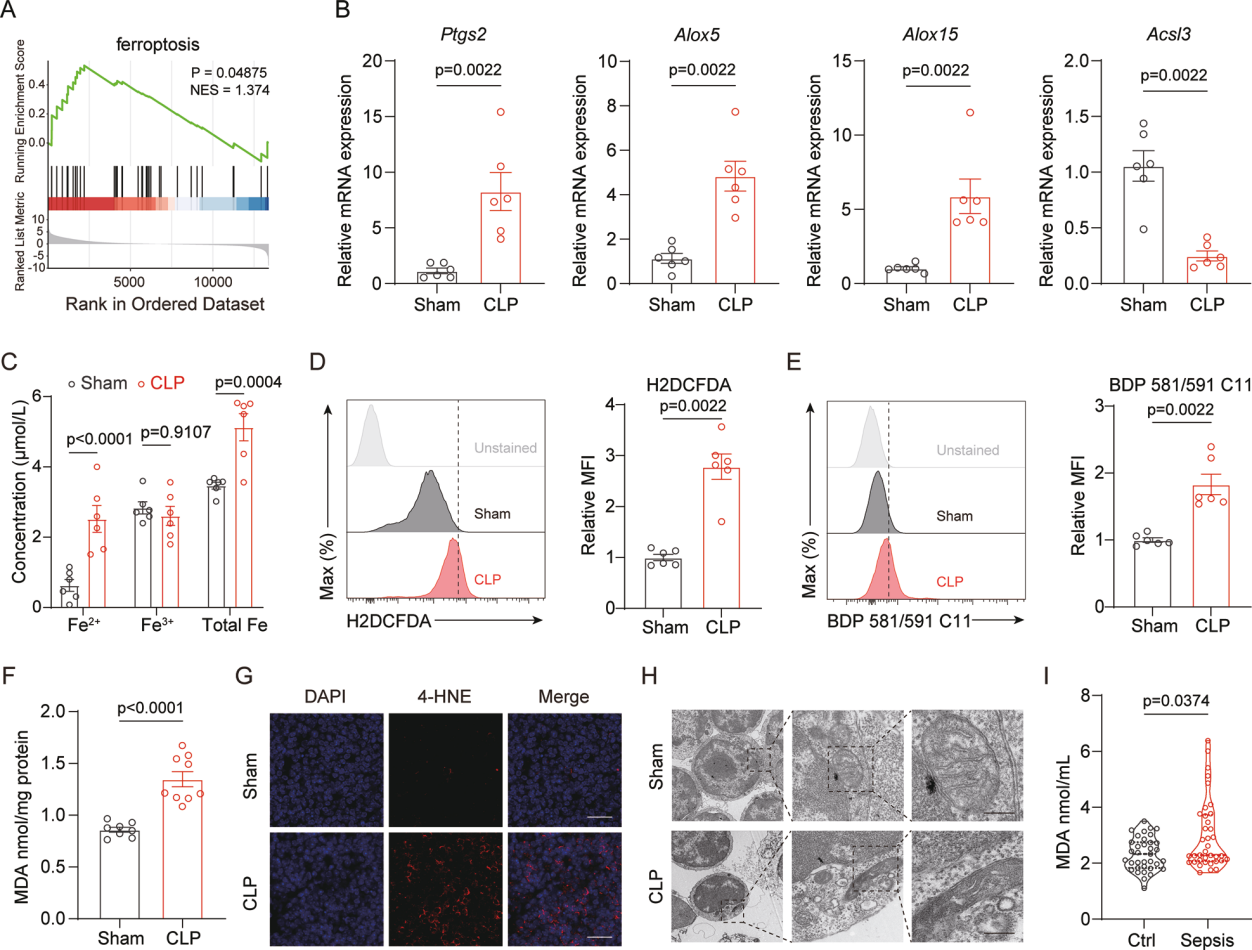
Sepsis-associated ATI and thymocyte death are linked to ferroptosis. **A** Gene set enrichment analysis (GSEA) plot showing significant enrichment of the ferroptosis pathway in thymocytes from CLP-treated mice (*n* = 4). **B** Relative mRNA expression levels of *Ptgs2, Alox15, Alox5*, and *Acsl3* in thymocytes from sham and CLP mice (*n* = 6). **C** Quantification of Fe²⁺, Fe³⁺, and total iron levels in thymocytes from sham and CLP mice (*n* = 6). **D** Flow cytometry analysis of ROS levels in thymocytes using H2DCFDA staining in thymocytes from sham and CLP mice, represented by flow cytometry histograms (left) and mean fluorescence intensity (MFI) quantification (right) (*n* = 6). **E** Lipid peroxidation levels measured via BDP 581/591 C11 staining in thymocytes from sham and CLP mice, represented by flow cytometry histograms (left) and MFI quantification (right) (*n* = 6). **F** MDA concentration in thymic tissue from sham and CLP mice (*n* = 8-9). **G** Representative immunofluorescence images showing 4-HNE staining in sham and CLP murine thymus (scale bar = 25 μm). **H** TEM images of thymocyte mitochondria, showing ferroptosis-associated damage in CLP mice compared to sham (scale bar = 200 nm). **I** MDA levels in plasma from pediatric sepsis patients and healthy controls (*n* = 35−38). Bars represent the means ± SEM.

In order to ascertain the potential mechanisms in the induction of acute thymic atrophy during sepsis, we collected thymocytes from CLP and sham-operated mice after 24 h post-surgery for subsequent RNA-seq analysis. A total of 3929 differential genes were detected, of which 2606 were upregulated and 1323 were downregulated (Fig. S4A–C). Increasing evidence suggests that ferroptosis appears to play a vital role in the development of sepsis-associated damage in organs, including lung, heart, kidney, intestinal, and liver [13–17]. However, the role of ferroptosis in ATI during sepsis is unexplored. Notably, RNA-seq results indicate that CLP-induced sepsis triggers significant alterations in ferroptosis-related genes, with GSEA revealing enrichment in ferroptosis pathways (Fig. 2A). We confirmed several key biomarkers of ferroptosis at the mRNA level, with RT-qPCR analysis in our experimental model showing marked upregulation of *Ptgs2, Alox5*, and *Alox15*, and downregulation of *Acsl3* in the thymus (Fig. 2B).

To further confirm the involvement of ferroptosis in sepsis-associated ATI, we conducted additional experiments focusing on ferroptosis indicators. We measured intracellular iron levels and found a significant increase in both Fe^2+^ and total iron ions (Fig. 2C). Moreover, we use H2DCFDA and BDP 581/591 C11 probes to label the levels of reactive oxygen species (ROS) and lipid peroxidation respectively. Flow cytometry results revealed that the ROS levels and lipid peroxidation indices within thymocytes were markedly elevated when compared to the sham group (Fig. 2D, E). In concordance, there was a notable augmentation in the malondialdehyde (MDA) content within the thymic tissue (Fig. 2F) and an enlarged area positive for 4-hydroxynonenal (4-HNE) (Fig. 2G), thereby signifying a comprehensive intensification of oxidative stress levels induced by sepsis. A notable characteristic of ferroptosis is the disruption of mitochondrial architecture. Through the application of transmission electron microscopy (TEM), we conducted a thorough examination of the mitochondrial morphology in thymocytes and identified that sepsis triggers mitochondrial irregularities, typified by a reduced or complete lack of mitochondrial cristae, a ruptured outer mitochondrial membrane and a smaller size as well (Fig. 2H). To verify the correlation between the pathophysiological process of sepsis and ferroptosis, we collected plasma samples from pediatric sepsis patients and control patients. Consistently, MDA levels in the plasma of sepsis patients were significantly elevated (Fig. 2I). These observations strongly suggest a significant correlation between the onset of ferroptosis and sepsis-associated ATI.

### Inhibition of ferroptosis ameliorates sepsis-associated ATI in vivo

**Fig. 3:**
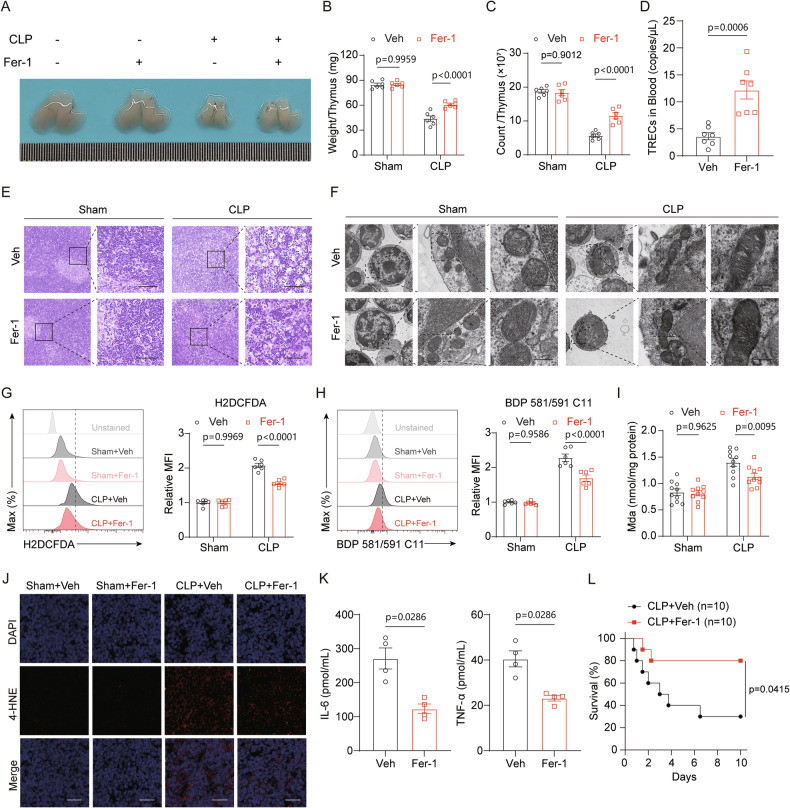
Inhibition of ferroptosis alleviates ATI. **A** Representative images of thymus morphology from sham and CLP mice treated with vehicle or Fer-1. **B**, **C** Quantification of thymus weight (**B**) and total thymocyte counts (**C**) in sham and CLP mice after Fer-1 administration (*n* = 6). **D** Quantification of thymic TRECs in peripheral blood from CLP mice following Fer-1 treatment (*n* = 7). **E** Representative HE-stained thymic sections from sham and CLP mice with vehicle or Fer-1 treatment (scale bar = 50 μm). **F** TEM images of thymocytes showing restoration of mitochondrial structure in Fer-1-treated CLP mice (scale bar = 200 nm). **G**, **H** Flow cytometric analysis of ROS production (**G**) and lipid peroxidation (**H**) in sham and CLP thymocytes after Fer-1 treatment (*n* = 6). **I** MDA levels in thymic tissue from sham and CLP mice with Fer-1 treatment (*n* = 10). **J** Immunofluorescence staining of 4-HNE in sham and CLP murine thymus treated with vehicle or Fer-1 (scale bar = 25 μm). **K** Plasma levels of IL-6 and TNF-α in CLP mice with and without Fer-1 treatment (*n* = 4). **L** Kaplan-Meier survival curves of CLP mice treated with vehicle or Fer-1 (*n* = 10). Bars represent the means ± SEM.

Next, we investigated whether blocked ferroptosis could ameliorate sepsis-associated ATI. Ferrostatin-1 (Fer-1), a potent and selective ferroptosis inhibitor, acts via a reductive mechanism to safeguard membrane lipids from damage, thereby preventing cell death [26]. We administered Fer-1 to track the characteristic alterations pertinent to ferroptosis within the thymus. The results indicated that Fer-1 significantly alleviated thymic injury, as evidenced by the lesser degree of reduction in thymus size, weight and cell counts (Fig. 3A–C), accompanied by a pronounced improvement in thymic output function (Fig. 3D) when compared to the vehicle-treated CLP group. Furthermore, Fer-1 treatment resulted in a significant increase in peripheral blood T-cell counts and a reduction in bacterial colony-forming units (CFUs) in the blood (Fig. S5A, B). Histological analysis using hematoxylin and eosin (HE) staining confirmed that the focal tissue damage within the thymus was reversed by Fer-1 during sepsis (Fig. 3E). TEM analysis revealed a recovery in both the number and structure of mitochondria in thymocytes following ferroptosis inhibition, consistent with the expected outcomes (Fig. 3F). Additionally, staining with H2DCFDA, BDP 581/591 C11, and 4-HNE of thymic tissue, together with the MDA levels, collectively indicated that Fer-1 effectively mitigated sepsis-induced oxidative stress (Fig. 3G–J). More critically, the administration of Fer-1 markedly reduced the serum levels of IL-6 and TNF-α, as well as mortality rates in CLP mice (Fig. 3K, L). Taken together, these data demonstrated that inhibition of ferroptosis can strongly ameliorate thymus homeostasis and improve mortality of sepsis.

### IDO1-mediated kynurenine accumulation exacerbates ferroptosis in thymocytes

**Fig. 4:**
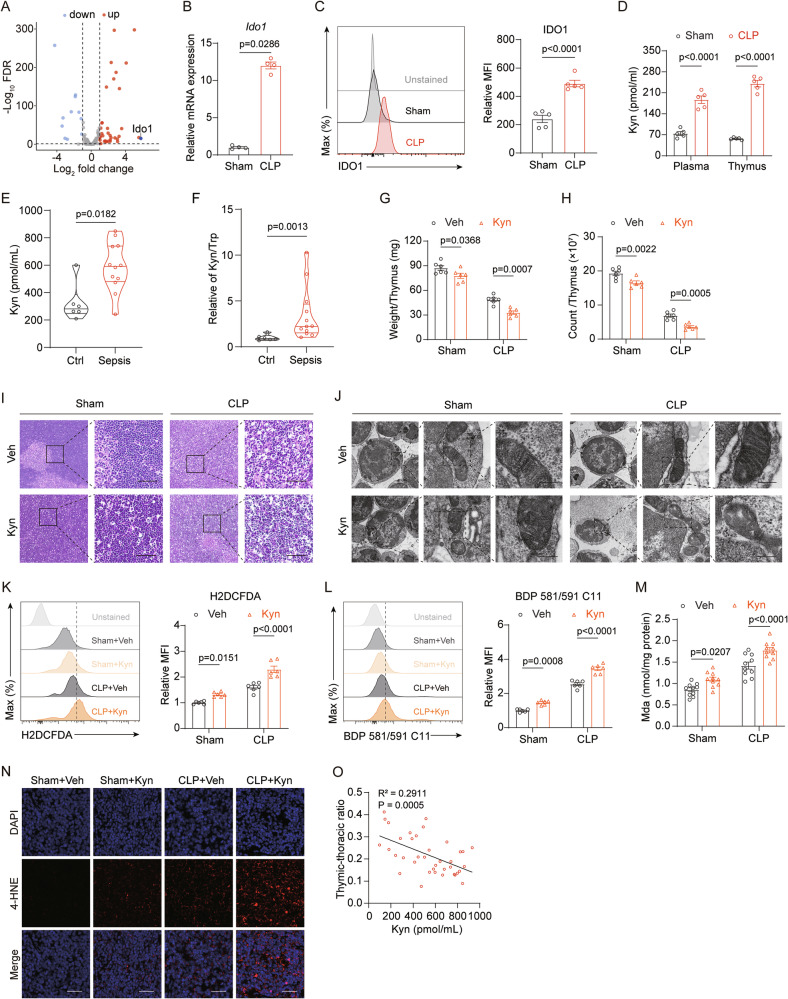
Thymic Kyn accumulation triggered by IDO1 exacerbates ferroptosis. **A** Volcano plot showing differentially expressed ferroptosis-driver genes in thymocytes from sham and CLP mice (*n* = 4) based on RNA-seq analysis. **B** Relative mRNA expression levels of *Ido1* in thymocytes from sham and CLP mice (*n* = 4). **C** Flow cytometry analysis of IDO1 protein expression in thymocytes from sham and CLP mice (*n* = 5). **D** Kyn levels in plasma and thymus of sham and CLP mice (*n* = 5). **E**, **F** Plasma Kyn (**E**) levels and Kyn/Trp (**F**) in sepsis patients (*n* = 12) versus healthy controls (HC) (*n* = 6). **G**, **H** Quantification of thymus weight (**G**) and total thymocyte counts (**H**) in sham and CLP mice treated with vehicle or Kyn (*n* = 6). **I** Representative HE-stained thymic sections from sham and CLP mice treated with vehicle or Kyn (scale bar = 50 μm). **J** TEM images of thymocytes showing mitochondrial damage in CLP mice after Kyn administration (scale bar = 200 nm). **K**, **L** Flow cytometry analysis of ROS production (**K**) and lipid peroxidation (**L**) in sham and CLP thymocytes after Kyn treatment (*n* = 6). **M** MDA concentration in thymic tissue from sham and CLP mice treated with Kyn (*n* = 10). **N** Immunofluorescence staining of 4-HNE in thymic sections from sham and CLP mice treated with vehicle or Kyn (scale bar = 200 nm). **O** Correlation analysis between plasma Kyn levels and the thymic-to-thoracic ratio (*n* = 38). Bars represent the means ± SEM.

Utilizing the FerrDb database, we compiled a compendium of driver genes implicated in ferroptosis (Supplementary Data S1). Subsequently, we conducted an overlap analysis with the differential gene expression profile of thymocytes from the CLP and Sham mice, thereby identifying a cohort of genes potentially involved in the ferroptosis induced by sepsis (Fig. S6). Notably, our findings revealed that *Ido1* is the most prominently upregulated driver gene associated with ferroptosis in sepsis-associated ATI (Fig. 4A). IDO1, characterized as a monoheme bound enzyme, is ubiquitously expressed across various human tissues and serves as the first rate-controlling enzyme within the tryptophan (Trp) metabolism via the Kyn pathway [27]. Exceeding 95% of Trp undergoes metabolic conversion through this pathway, culminating in the production of several distal metabolites, including Kyn. Prior studies have elucidated that the expression levels of IDO1 are markedly augmented in response to inflammatory or infectious stimuli, as compared to baseline physiological conditions [28]. In this context, the findings derived from RT-qPCR and flow cytometric analyses have served to validate a significant upsurge in both the mRNA and protein concentrations of IDO1 within the thymocytes, occurring 24 h after CLP surgery (Fig. 4B, C). ELISA analysis further revealed that sepsis induced a localized accumulation of Kyn in both the bloodstream and thymus (Fig. 4D), providing additional evidence of increased IDO1 activity. The enzymatic activity of IDO1 is often assessed by measuring the ratio of Trp to Kyn concentrations. To determine whether IDO1 activity and Kyn levels are elevated in pediatric sepsis patients, we measured plasma Kyn and Trp concentrations in sepsis patients and healthy controls. The results showed that the Kyn levels was significantly higher in septic children compared to controls, with Kyn/Trp ratio markedly elevated (Fig. 4E, F).

To determine whether ferroptosis influences IDO1/Kyn levels in thymocytes during sepsis, we measured IDO1 expression and Kyn production in CLP mice treated with the ferroptosis inhibitor Fer-1. We found that Fer-1 treatment did not significantly affect *Ido1* mRNA (Fig. S7A), IDO1 protein levels (Fig. S7B), or Kyn concentrations in plasma and thymic tissue (Fig. S7C). These results indicate that ferroptosis does not regulate IDO1 expression or Kyn production, further supporting the hypothesis that the IDO1/Kyn pathway functions upstream of ferroptosis activation in sepsis.

Given the absence of direct evidence indicating that Kyn exacerbates ferroptosis and ATI, supplementation with Kyn was administered in septic mice to simulate the endogenous increase of Kyn levels. Kyn treatment accelerated the progression of thymic atrophy, evidenced by significant reductions in thymus volume, quality, and cell population (Fig. S8; Fig. 4G, H). Histopathological evaluation revealed exacerbated injury to the thymic cortex and medulla following Kyn supplementation (Fig. 4I). Furthermore, TEM elucidated that there was severe disruption to the mitochondrial cristae (Fig. 4J). As anticipated, the profoundly elevated levels of Kyn significantly aggravated the thymic oxidative stress response and lipid peroxidation process (Fig. 4K-N). Interestingly, even in the sham surgery control group, excessive Kyn led to discernible tissue damage and oxidative stress within the thymus. Inhibiting ferroptosis rescued the effect of Kyn administration on thymic involution (Fig. S9). To examine the correlation between Kyn levels and thymus size in pediatric sepsis patients, we recruited 38 septic children, recorded the TT-ratio, and collected plasma for Kyn analysis. Correlation analysis revealed a significant negative correlation between Kyn levels and the TT-ratio (Fig. 4O). Consequently, it is postulated that the excessive activation of the IDO1 in sepsis is a result of inflammatory processes, with the accumulation of Kyn being a pivotal element in the onset of ferroptosis.

### IDO1 inhibition mitigates ferroptosis in thymocytes and improves survival in sepsis

**Fig. 5:**
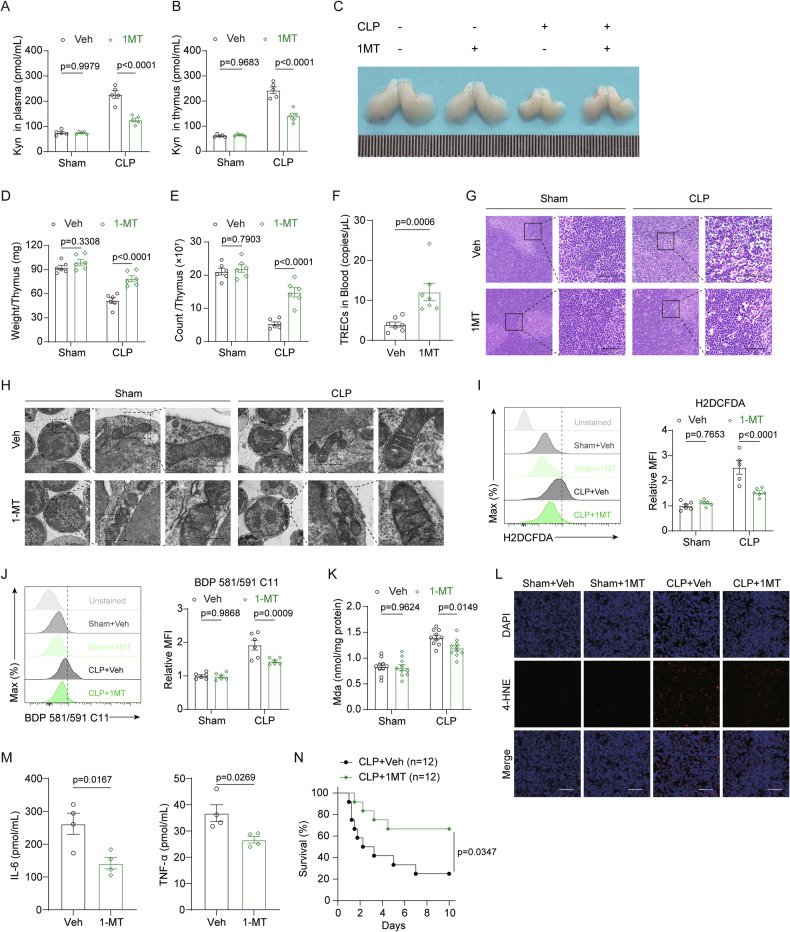
IDO1 inhibitor 1-MT curtails Kyn-AhR signaling cascade to mitigate thymic ferroptosis and sepsis survival. **A**, **B** Kyn levels in plasma (**A**) and thymus (**B**) of sham and CLP mice after 1-MT administration (*n* = 5). **C**−**E** Representative images of thymus morphology (**C**), and quantification of thymus weight (**D**) and thymocyte counts (**E**) in sham and CLP mice treated with 1-MT (*n* = 6). **F** Quantification of thymic TRECs in peripheral blood from CLP mice treated with 1-MT (*n* = 7). **G** Representative HE-stained thymic sections from sham and CLP mice treated with 1-MT (scale bar = 50 μm). (**H**) TEM images showing mitochondrial structure in thymocytes from CLP mice after 1-MT treatment (scale bar = 200 nm). **I**, **J** Flow cytometry analysis of ROS production (**I**) and lipid peroxidation (**J**) in thymocytes from sham and CLP mice treated with 1-MT (*n* = 6). **K** MDA levels in thymic tissue from sham and CLP mice treated with 1-MT (*n* = 10). **L** Immunofluorescence staining of 4-HNE in thymic sections from sham and CLP mice treated with 1-MT (scale bar = 25 μm). **M** Plasma levels of IL-6 and TNF-α in CLP mice treated with 1-MT (*n* = 4). **N** Kaplan-Meier survival curves of CLP mice treated with vehicle (Veh) or 1-MT (*n* = 12). Bars represent the means ± SEM.

To evaluate the direct impact of Kyn restriction on the recovery of sepsis-associated ATI, we inhibited Kyn synthesis by administering an IDO1 inhibitor, 1-methyltryptophan (1-MT). Plasma and thymic Kyn concentrations were measured 24 h post-surgery. As expected, 1-MT treatment effectively suppressed Kyn levels in both the thymus and systemically (Fig. 5A, B). Similar to the therapeutic benefits observed with Fer-1, 1-MT demonstrated remarkable efficacy in restoring thymic size, weight, cell count, and TRECs levels in the blood (Fig. 5C-F). Furthermore, 1-MT administration led to a marked increase in peripheral blood T-cell counts and a reduction in bacterial CFUs in circulation (Fig. S5C, D). HE staining and TEM imaging further revealed that IDO1 inhibition plays a crucial role in preserving thymic structural integrity and mitigating mitochondrial damage during early sepsis (Fig. 5G, H). Moreover, 1-MT treatment was highly effective in limiting ROS production and reducing lipid peroxidation, thereby preventing ferroptosis in the thymus (Fig. 5I–L). Importantly, 1-MT administration significantly decreased the secretion of inflammatory cytokines (Fig. 5M) and substantially reduced long-term mortality rates associated with polymicrobial sepsis (Fig. 5N). These findings suggest that IDO1 inhibition effectively mitigates thymic ferroptosis and improves survival outcomes in sepsis.

To assess whether co-administration of 1-MT and Fer-1 provides synergistic benefits in treating thymic involution, we conducted additional experiments. Our results indicate that the combined administration of both inhibitors does not confer any additional therapeutic advantage beyond their individual effects (Fig. S10). These findings indicate that 1-MT and Fer-1 do not act synergistically in mitigating thymic involution, supporting our hypothesis that these inhibitors function within the same signaling pathway rather than through independent mechanisms.

### Thymic integrity is essential for therapeutic efficacy of ferroptosis inhibition in Sepsis

While ferroptosis inhibition demonstrates systemic therapeutic potential in sepsis, its tissue-specific mechanisms remain undefined the involvement of multiple organs in ferroptotic processes [13–17]. To dissect thymic contributions, we implemented a thymectomy-based experimental paradigm coupled with Fer-1 or 1-MT (Fig. S11A). Thymectomized septic mice exhibited profound depletion in peripheral T-cell counts and uncontrolled bacterial dissemination compared to sham controls (Fig. S11B, C), recapitulating clinical thymectomy-associated immunosuppression. Crucially, both Fer-1 and 1-MT lost therapeutic efficacy in thymectomized animals, failing to restore T-cell homeostasis or microbial clearance. While thymectomy markedly exacerbated mortality, the attenuated survival benefits in treated thymectomized mice versus intact-thymus counterparts (Fig. S11D) confirm that thymic targeting is prerequisite for optimal intervention outcomes. These findings collectively demonstrate that thymic ferroptosis critically contributes to sepsis mortality, though additional mechanisms may synergize. This thymus-dependent mechanism aligns with clinical evidence linking thymus removal to heightened mortality risk [6], highlighting the thymus as a critical mediator of ferroptosis-driven pathology in sepsis.

### Kyn-activated AhR promotes the lipid peroxidation of thymocytes in sepsis

**Fig. 6:**
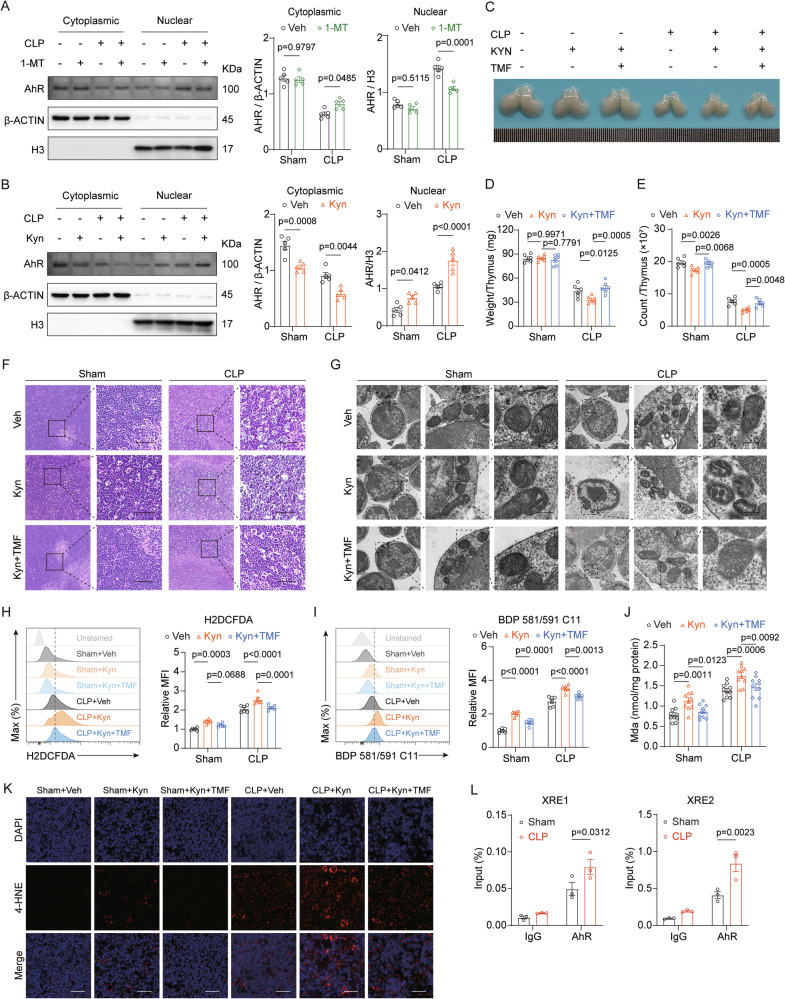
Kyn-activated AhR promotes lipid peroxidation in thymocytes of CLP mice. **A** AhR expression in the nucleus and cytoplasm of thymocytes from sham and CLP mice after 1-MT treatment compared with vehicle (*n* = 5). **B** AhR expression in the nucleus and cytoplasm of thymocytes from sham and CLP mice after Kyn treatment compared with vehicle (*n* = 5). **C**−**E** Representative images of thymus morphology (**C**), and quantification of thymus weight (**D**) and thymocyte counts (**E**) in sham and CLP mice after treatment with Kyn and AhR inhibitor TMF, compared to Kyn or vehicle alone (*n* = 6). **F** Representative HE staining of thymocytes from sham and CLP mice treated with Kyn and TMF, compared to Kyn or vehicle alone (scale bar = 50 μm). **G** Representative TEM images of thymocytes from sham and CLP mice treated with Kyn and TMF, compared to Kyn or vehicle alone (scale bar = 200 nm). **H**, **I** Flow cytometry analysis of ROS production (**H**) and lipid peroxidation (**I**) in thymocytes from sham and CLP mice treated with Kyn and TMF, compared to Kyn or vehicle alone (*n* = 6). **J** MDA levels in thymic tissue from sham and CLP mice treated with Kyn and TMF, compared to Kyn or vehicle alone (*n* = 10). **K** Immunofluorescence staining of 4-HNE in thymic sections from sham and CLP mice treated with Kyn and TMF, compared to Kyn or vehicle alone (scale bar = 25 μm). **L** Thymocytes were isolated from sham and CLP mice 24 h post-surgery. ChIP analysis was performed to assess AhR binding at XRE sites within the *Pla2g4a* promoter region, with IgG serving as a negative control (*n* = 3). Bars represent the means ± SEM.

It has been established that Kyn functions as an archetypal endogenous ligand for the AhR, an integral member of the bHLH-PAS transcription factor family [29]. Our previous research demonstrated that AhR activation is associated with the exacerbation of ferroptosis [30]. To determine whether the downstream signaling pathways of IDO1-derived Kyn in ferroptosis depend on AhR, we performed western blot analysis. The results revealed that the total levels of AhR in thymocytes were unaffected (Fig. S12A). However, studies have shown that Kyn activates AhR, promoting its nuclear translocation, where it forms a complex with the AhR nuclear translocator (ARNT) and binds to gene promoters, thereby stimulating gene transcription. We confirmed increased expression of *Cyp1a1*, a marker of AhR transcriptional activation, using RT-qPCR (Fig. S12B). Nuclear and cytoplasmic fractionation of thymocytes further revealed that, compared to the control group, AhR levels were elevated in the nucleus and reduced in the cytoplasm, indicating enhanced nuclear translocation and activation (Fig. S12C). Moreover, inhibition of Kyn synthesis with 1-MT decreased AhR nuclear translocation (Fig. 6A), while exogenous Kyn administration further increased AhR nuclear translocation (Fig. 6B), providing robust evidence that AhR activation is driven by Kyn.

To further investigate the role of AhR in Kyn-induced ferroptosis, we administered the potent AhR antagonist, 6,2’,4’-trimethoxyflavone (TMF), to the Kyn-supplemented CLP group to inhibit AhR-mediated gene induction. As expected, TMF treatment significantly improved the adverse effects caused by Kyn supplementation, notably enhancing thymic size, weight, cell counts, tissue integrity, and ultrastructural architecture. (Fig. 6C-G). Additionally, TMF treatment effectively suppressed Kyn-mediated lipid peroxidation, as confirmed by reduced levels of ROS, lipid peroxidation markers, MDA, and 4-HNE (Fig. 6H-K). These findings support the notion that AhR activation by Kyn promotes lipid peroxidation, thereby exacerbating thymic ferroptosis in sepsis.

To investigate how AhR regulates ferroptosis-related genes in thymocytes, we performed ChIP assays. Bioinformatics analysis identified AhR binding motifs (-GCGTG-) within the promoter region of *Pla2g4a*, a known regulator of ferroptosis [30]. ChIP assays revealed a significant enrichment of AhR binding at the predicted xenobiotic response elements (XREs) in CLP-treated mice compared to sham controls. Specifically, increased AhR occupancy was detected at XRE1 (-1182 bp to -1178 bp relative to the transcription start site) and XRE2 (-1002 bp to -998 bp), suggesting that AhR may directly regulate transcription at these sites (Fig. 6L). Moreover, we found that kynurenine could enhance CLP-induced *Pla2g4a* expression (Fig. S13), further supporting its transcriptional regulation by AhR. These findings indicate that AhR directly modulates *Pla2g4a* expression, thereby contributing to ferroptosis activation in thymocytes.

## Discussion

In this study, we investigated the role of ferroptosis in ATI during sepsis. Our results showed that sepsis-induced ATI was characterized by a marked reduction in thymic size, cellularity, and T cell counts, and persisted for an extended period post-infection. Ferroptosis was identified as a significant driver of this thymic atrophy, evidenced by significant oxidative stress, mitochondrial damage, and increased lipid peroxidation in thymocytes. We found that upregulation of IDO1 led to the accumulation of Kyn, which subsequently activated the AhR and drove ferroptosis in thymocytes. Inhibition of IDO1 using 1-MT successfully reversed thymic ferroptosis, restored thymic function, and improved survival in septic mice.

The thymus plays a crucial role in the immune system. Its proper function is essential for maintaining both the quality and quantity of the naive T cell pool. In addition to chronic thymic atrophy caused by aging, external factors such as infections, radiation, environmental toxins, malnutrition, and excessive hormonal exposure can trigger ATI [24, 31]. Research has shown that infection-induced ATI reduces TCR repertoire diversity, accelerates the aging of naive T cells, and disrupts T cell subset balance, significantly impairing the host’s antigen response [10, 32]. Although the thymus exhibits some self-repair capabilities, recent studies have highlighted that acute injury can lead to the accumulation of age-related thymic epithelial cells, disrupting the regenerative pathways of the thymus [33]. Currently, little is known about the mechanisms that drive thymic regeneration and functional recovery.

The essence of sepsis lies in infection-triggered immune dysregulation, involving ATI and T lymphocyte exhaustion [34]. In our study, we found that peripheral T lymphocyte counts were lower in deceased pediatric sepsis patients, suggesting that T cell depletion may be a critical risk factor for poor outcomes. Additionally, the thymus size in sepsis patients was significantly reduced compared to healthy controls, indicating that sepsis accelerates ATI in children. Further correlation analyses revealed a significant positive relationship between thymic size and peripheral blood total T cells, CD4^+^ T cells, and CD8^+^ T cells, as well as a significant negative correlation with inflammatory markers, such as CRP, IL-6, and PCT. These findings suggest that the excessive inflammatory response in pediatric sepsis may contribute to ATI and further deplete peripheral T cells, thereby increasing the risk of death. The recovery timeline for ATI following sepsis remains largely unclear. In our mouse model of sepsis, we observed that thymus size and TREC levels in surviving mice had not returned to normal even 10 days after CLP (approximately equivalent to 400 days in humans [35]). Similarly, a study on COVID-19 patients showed persistent CD8^+^ T cell depletion up to 12 months post-infection [36]. Besides, small thymus size in early infancy is a strong risk factor for mortality [37, 38]. Given the association between ATI and T cell depletion in pediatric sepsis with increased mortality risk, and the crucial role of thymic function in establishing the immune barrier in children, our study aims to investigate the triggers of sepsis-related ATI. This is particularly important in light of the current lack of effective therapeutic strategies to restore thymic function.

Cell death is a key feature of sepsis-associated ATI, potentially involving various forms of cell death, such as previously reported apoptosis [9]. By administering Fer-1 to septic mice, we found that blocking ferroptosis effectively alleviates ATI, suggesting that ferroptosis plays a crucial role in ATI. Ferroptosis has been extensively implicated in sepsis-related organ damage. Inhibiting ferroptosis can partially reduce parenchymal cell damage in various sepsis models, such as cardiomyocytes, pulmonary epithelial cells, renal tubular cells, and hepatocytes, thereby ameliorating associated organ injury [13–16]. Additionally, ferroptosis has also been identified in immune cells, including CD4 T cells, macrophages, and dendrite cells during sepsis [18–20]. Typically, ferroptosis occurs rapidly, particularly under conditions of iron overload [39]. Our experimental results demonstrated significant increases in both Fe^2+^ and total iron levels within thymocytes. Previous studies indicate that ferroptotic cells exhibit immunogenic properties, which can amplify the inflammatory cascade and contribute to further cell death [12]. In our mouse model of sepsis, we observed rapid sepsis-induced ATI, evidenced by significant thymic atrophy and widespread thymocyte death within 24 h of CLP. Transcriptomic analysis identified significant changes in ferroptosis-related genes, such as *Ptgs2, Alox5, Alox15, Acsl3*, and others. RNA-seq analysis of thymocytes from both sham-operated and septic mice, coupled with the administration of a ferroptosis inhibitor in vivo, confirmed that ferroptosis plays a central role in sepsis-induced ATI. To the best of our knowledge, this study presents new evidence indicating the presence of ferroptosis in thymocytes during sepsis. To further elucidate the drivers of thymocyte ferroptosis, we compared the differentially expressed genes from our transcriptomic analysis with ferroptosis driver genes listed in the FerrDb database. This approach identified IDO1 as a key driver of thymocyte ferroptosis.

IDO1 is a pivotal immunoregulatory enzyme that plays a critical role in Trp metabolism, catalyzing the first rate-limiting step in the Trp-Kyn pathway. The expression of IDO1 can be upregulated by various inflammatory stimuli, such as IL-6, TNF-α, and IFN-γ [40]. IDO1 has been extensively studied in the context of cancer, where its overexpression is often linked to immune evasion by tumor cells, potentially through mechanisms such as inhibiting T cell activation, promoting T cell apoptosis, and expanding regulatory T cell (Treg) populations [41]. Moreover, IDO1 has been implicated in promoting oxidative stress and apoptosis during myocardial infarction and renal ischemia-reperfusion injury [42, 43]. In the context of sepsis, increased IDO1 expression is believed to contribute to sepsis-associated immunosuppression, broadly affecting various immune cell populations, including T cells, B cells, monocytes, and neutrophils [28]. However, there remains limited understanding of IDO1’s regulatory role in thymocytes during sepsis.

Increased IDO1 activity primarily manifests as local Trp depletion and Kyn accumulation. Once IDO1 is activated and initiates the production of Kyn, the AhR-IL-6-signal transducer and activator of transcription 3 (STAT3) loop can sustain the continuous activation of IDO1 [28]. In our study, we found significant increases in Kyn levels in both the thymus and plasma during the acute phase of sepsis in mice. Kyn, known for its pro-oxidant properties, exacerbates oxidative stress by promoting ROS generation and lipid peroxidation [44–46]. Importantly, we also found significantly elevated Kyn levels in the plasma of septic children compared to healthy controls. Previous research has linked increased Kyn levels in sepsis patients to systemic hypotension [47], potentially due to endothelial dysfunction caused by Kyn accumulation. Our experiments demonstrated that inhibiting IDO1 effectively reduced Kyn synthesis, thereby reversing thymocyte ferroptosis and improving long-term survival in septic mice. Furthermore, IDO1 inhibition alleviated systemic inflammation, as indicated by decreased IL-6 and TNF-α levels in the plasma of treated mice. In pediatric sepsis patients, plasma Kyn levels were significantly negatively correlated with thymus-to-thorax ratios and T cell counts, providing additional evidence of the association between Kyn accumulation and ATI.

The AhR is a ligand-activated transcription factor that belongs to the bHLH-PAS (basic helix-loop-helix Per-Arnt-Sim) protein family. Kyn functions as an AhR agonist, promoting AhR activation and subsequent nuclear translocation to regulate the transcription of target genes. We found that exogenous administration of Kyn in both sham and septic mice enhanced the translocation of AhR into the nucleus of thymocytes. Previous studies have demonstrated that the AhR agonist 2,3,7,8-tetrachlorodibenzo-p-dioxin (TCDD) can induce thymic atrophy independently of thymic epithelial cells and Fas/Fas ligand signaling [48, 49]. In our previous research, we also showed that AhR activation in airway epithelial cells contributes to ferroptosis by increasing the transcription of *Pla2g4a* [30]. In this study, we observed that administration of the AhR inhibitor TMF reversed Kyn-induced ferroptosis in thymocytes, indicating that AhR activation is involved in thymocyte ferroptosis. Using a transcription factor target gene prediction platform, we identified *Pla2g4*, *Alox5*, and *Alox15* as key genes potentially involved in AhR-driven ferroptosis by intersecting AhR target genes, differentially expressed genes from RNA-seq, and ferroptosis driver genes from the FerrDb database. Furthermore, we confirmed by ChIP assay that *Pla2g4a* is a key target gene of AhR, critically involved in thymocyte ferroptosis.

This study has inherent limitations. Culturing thymocytes in vitro remains challenging due to the complex nature of replicating the thymic microenvironment, the requirements for multi-stage development, the demand for specific cytokines, and the difficulty in constructing three-dimensional structures that maintain long-term functionality [50]. Consequently, we restricted our experimental validation to in vivo drug administration, focusing on findings at the level of the entire thymocyte population. Future advances in three-dimensional thymic organoid technologies, as well as optimized cytokine and signaling regulation systems, could enhance culture efficiency and provide deeper insights into sepsis-induced ATI.

In summary, our study demonstrates that T lymphocyte exhaustion is closely associated with sepsis-induced ATI. Mechanistically, our findings reveal that inflammatory signals upregulate IDO1 expression, which drives Kyn accumulation, leading to lipid oxidation-related gene transcription via activation of the AhR and ultimately resulting in thymocyte ferroptosis during sepsis. Targeting the IDO1/Kyn/AhR axis may provide a promising therapeutic strategy to restore T cell immunity and improve outcomes for patients with sepsis.

## Materials and Methods

### Ethics approval and consent to participate

The study was conducted in compliance with the Declaration of Helsinki and received approval from the Ethics Committee of the Children’s Hospital of Fudan University (2021431). Informed written consent was obtained from all guardians as well. Animals were cared for and handled in accordance with the Guide for Care and Use of Laboratory Animals. All experiments were approved by the Animal Study Committee of the Children’s Hospital of Fudan University.

### Participants and clinical data

Pediatric patients were recruited from the Pediatric Intensive Care Unit (PICU) at the Children’s Hospital of Fudan University between January 2022 and June 2024. Participants diagnosed with sepsis, aged between 28 days and 13 years, were included. Patients with a history of hematological diseases, malignant solid tumors, autoimmune diseases, or immunodeficiency disorders were excluded. Children with accident trauma, but without infectious diseases or tumors, who underwent chest computed tomography (CT) examinations were recruited as controls. Peripheral blood was collected within 24 h of admission and the plasma was isolated immediately and stored at -80 °C until further analysis. Clinical data were recorded at 24 h after admission, including sex, age, white blood cell count (WBC), CRP, PCT, IL-6, absolute T cell count in peripheral blood, Pediatric Sequential Organ Failure Assessment (p-SOFA) score, and chest CT images. Additionally, microbiological findings, ventilation, use of extracorporeal membrane oxygenation (ECMO), hemopurification, and mortality within 28 days of admission was documented. The characteristics of sepsis and control group were shown in Table S1. The TT-ratio, measured at the level of the aortopulmonary window, serves as a marker of relative thymus size, characterized by the ratio of thymic thickness to the distance between the sternum and vertebral body [51, 52]. The measurements were conducted using a Philips IntelliSpace IX Workstation (Philips Healthcare, Amsterdam, NL).

### Animals

Male wild-type C57BL/6 J mice (4 weeks old, 11−13 g body weight) were purchased from Shanghai Shengchang Biotechnology Co., Ltd, Shanghai, China. All animals were housed at 25 °C with a 12 h light/dark cycle under specific pathogen-free conditions, with free access to food and water, and were acclimated to the environment for 48 h prior to surgery. In in vivo experiments, sample sizes were determined based on prior similar studies and our experimental experience, ensuring reproducible and statistically meaningful results.

### Polymicrobial sepsis model

Polymicrobial sepsis was induced in mice using the CLP procedure [53]. Briefly, a 5 mm midline abdominal incision was made, and the cecum was ligated with 4–0 silk at two-thirds of the distance between the distal pole and the ileocecal valve (Fig. S2A), then perforated once with a 22-gauge needle. A small amount of feces was extruded into the abdominal cavity. The abdominal wall and skin were closed in two layers using 6-0 and 4–0 silk sutures, respectively. Sham-operated mice underwent identical procedures, excluding cecal ligation and puncture. All mice received pre-warmed physiological saline solution (0.5 mL, 37 °C) subcutaneously for postoperative fluid resuscitation and were kept warm until fully recovered from anesthesia. Post-surgical mice were housed in a temperature-controlled room (25 °C) with free access to food and water. The mortality rate of CLP mice within 10 days, excluding procedural failures, was ~70% (Fig. S2B).

### Thymectomy procedure

The thymic ablation protocol was performed by meticulous surgical dissection. Following thoracic depilation, mice were positioned in supine orientation under anesthesia. A midline skin incision was made at the superior border of the sternum, followed by blunt dissection through the underlying tissues to expose the sternum. The upper sternum was then carefully incised along the midline. Pretracheal muscles were gently separated to expose the upper poles of the thymus. Each thymic lobe was grasped at the upper pole and gently retracted outward. Once fully exposed, the thymus was promptly excised. The sternum and skin were then closed in layers using sutures. Sham controls underwent identical exposure without thymic manipulation. Post-surgical mice were housed in a temperature-controlled room (25 °C) with free access to food and water. All animals completed a 7 day convalescence period prior to subsequent CLP surgery.

### Isolation of thymocytes

Mice were euthanized with CO_2_, and the thymus was quickly isolated and placed in ice-cold phosphate-buffered saline (PBS). The thymus was then cut into small pieces (-2 mm³) and immediately homogenized. The suspension was passed through a 40 µm filter to obtain a thymocyte single-cell suspension, while remnants mainly consisted of thymic stromal cells. The thymocyte suspensions were used for the following experiments.

### Flow cytometry

Single-cell suspensions were obtained from the thymus or peripheral blood samples. Peripheral blood cells were counted using Precision Count Beads™ (BioLegend, San Diego, CA, USA) in a lyse-no-wash whole blood assay following the manufacturer’s instructions. And samples were stained with the following antibodies: FITC anti-mouse CD45.2 Antibody (BioLegend), PE/Cyanine7 anti-mouse CD4 Antibody (BioLegend), PE anti-mouse CD8a Antibody (BioLegend), PE/Cyanine5 anti-mouse/human CD44 Antibody (BioLegend), FITC anti-mouse CD62L Antibody (BioLegend) and Alexa Fluor® 647 anti-IDO1 Antibody (BioLegend). PI staining (BD Biosciences, San Jose, CA, USA) was used for dead cell counting. The volumes and concentrations of antibodies or reagents were used according to the manufacturer’s instructions. Samples were analyzed using either a FACSDiva™ flow cytometer (BD Biosciences) or a CYTEK™ NL-1000 flow cytometer (Cytek Biosciences, Silicon Valley, CA, USA). Data were analyzed using FlowJo v10.8.1 software (Tree Star, Inc., Ashland, OR, USA).

### TRECs quantification

Peripheral blood samples were collected, and genomic DNA was extracted using the TIANamp Blood DNA Kit (Tiangen Biotech, Beijing, China). Absolute TRECs and internal control TCRA levels in genomic DNA were quantified using the AccuONE-300 Integrated digital PCR system (Zhenzhun Bio., Shanghai, China). Primers and probe are listed in the Table S2. Absolute TRECs counts in peripheral blood were calculated based on absolute cell counts (Fig. S14).

### RNA sequencing

Thymocytes were isolated from sham and CLP mice on day 1 post-surgery. RNA was extracted and purified using Trizol, and its purity and concentration were measured with a NanoDrop 2000 spectrophotometer. RNA integrity was checked with an Agient 2100. The mRNA was fragmented, reversely transcribed, and processed into a library for sequencing. Sequencing was performed using the Illumina NovaSeq 6000 in PE 150 mode, with data analyzed via BMKCloud (www.biocloud.net), provided by Biomarker Technologies, Beijing, China. The RNA sequencing data were deposited in the GEO database (accession number GSE280678).

### Real-time quantitative PCR

Real-time quantitative PCR (RT-qPCR) was used to measure mRNA levels in thymocytes. Total RNA was isolated using RNAiso Plus (Takara, Kusatsu, Japan), and cDNA was synthesized using the PrimeScript RT Master Mix kit (Takara). Target gene expression levels were quantified using TB Green Premix Ex Taq (Takara, RR420L) in the LightCycler® 480 RT-qPCR system (Roche, Basel, Switzerland). Primers are listed in the Table S2.

### Ionic iron detection

The levels of Fe^2+^ and Fe^3+^ were measured using a Iron Assay Kit (Dojindo, Kumamoto, Japan) according to the instructions provided.

### ROS detection

ROS generation was measured using H2DCFDA probe (MCE, Monmouth Junction, NJ, USA). Single-cell suspensions were obtained from the thymus. Samples were incubated with H2DCFDA dye (final concentration of 5 μM) in a constant temperature incubator at 37 °C for 20 min, protected from light. After washing twice with pre-chilled PBS (4 °C), fluorescence intensity was measured using flow cytometry (Ex/Em = 488/525 nm).

### Lipid peroxidation detection

Lipid peroxidation was measured using BDP 581/591 C11 probe (Dojindo). BDP 581/591 C11 dye was prewarmed at 37 °C before adding it to the thymocyte single-cell suspension. The samples were incubated with BDP 581/591 C11 dye in a constant temperature incubator at 37 °C for 30 min, protected from light. After washing the cells twice with pre-chilled PBS (4 °C), use a flow cytometer to detect fluorescence intensity at Ex/Em = 488/525 nm.

### MDA assay

MDA, a product of lipid peroxidation, was measured in plasma samples from children and thymus tissue from mice using an MDA assay kit, following the manufacturer’s instructions (Nanjing Jiancheng, Nanjing, China).

### HE staining

Thymus samples were fixed in 4% paraformaldehyde, dehydrated, and embedded in paraffin. Sections were deparaffinized with xylene, rehydrated through graded ethanol, and stained with hematoxylin and eosin.

### Immunofluorescence

Tissue sections were antigen-retrieved using citrate buffer and heating, blocked with 10% goat serum, and incubated with anti-4 hydroxynonenal antibody (abcam, Cambridge, UK, 1:200) overnight at 4 °C. After washing, sections were incubated with Cy3 conjugated Goat Anti-Rabbit IgG (H + L) (Servicebio, Wuhan, China, 1:300) in the dark at room temperature for 1 h. Then, slides were mounted using an Antifade Mounting Medium with DAPI (Beyotime, Shanghai, China) for nuclear staining.

### TEM assays

Obtain the thymocyte single-cell suspension according to the above procedure and quickly fixed in 2.5% glutaraldehyde (Servicebio, G1102). Thymocytes were fixed in the dark for 2 h at room temperature and then transferred to 4 °C for further storage. Mitochondrial ultrastructure was observed under TEM to determine the occurrence and severity of ferroptosis.

### In vivo administration of Fer-1, Kyn, TMF or 1-MT

Mice in the sham and CLP groups were administered intraperitoneal injections of Ferrostatin-1 (Fer-1, 10 mg/kg, MCE), Kynurenine (Kyn, 300 mg/kg, MCE), 6,2’,4’-Trimethoxyflavone (TMF, 5 mg/kg, MCE), or 1-Methyltryptophan (1-MT, 150 mg/kg, Sigma, St. Louis, MO, USA) at specific time points as outlined in Fig. S15. Fer-1 and Kyn were given 2 h before CLP, while TMF was administered both 12 h before and after CLP. 1-MT was injected 12 h before and 12 h after CLP. Control mice received equivalent volumes of vehicle. The animals were sacrificed 24 h after CLP or monitored to plot the survival curve over 10 days.

### Western blotting

Whole-cell lysates were prepared using RIPA Lysis Buffer (Beyotime) supplemented with Halt Protease Inhibitor Cocktail (Thermo Fisher Scientific, Waltham, MA, USA). After ultrasonic disruption on ice, the lysates were clarified by centrifugation at 12,000 g for 10 min at 4 °C. Nuclear and cytoplasmic proteins were extracted following the protocol of the Nuclear and Cytoplasmic Protein Extraction Kit (Beyotime). Protein concentrations were determined using the BCA method, and the protein samples were stored at -80 °C. Protein extracts or immunoprecipitated complexes were resolved by SDS-PAGE Gel Quick Preparation Kit (Beyotime) and transferred onto PVDF membranes. Membranes were blocked with Protein Free Rapid Blocking Buffer (Epizyme Biomedical Technology, Guangzhou, China) for 20 min at room temperature. Following blocking, membranes were incubated overnight at 4 °C with the following primary antibodies: AhR antibody (Enzo Life Sciences, Farmingdale, NY, USA, 1:1000), β-Actin Antibody (CST, Danvers, MA, USA, 1:1000), and Histone H3 Antibody (CST, 1:1000). After thorough washing with TBST, blots were incubated for 1 h at room temperature with Goat Anti-Rabbit IgG-HRP (Abmart, Shanghai, China, 1:5000). Proteins were detected using an enhanced chemiluminescence system, bands were visualized with the LAS-4000IR system, and band intensities were quantified using ImageJ software.

### Enzyme linked immunosorbent assay (ELISA)

ELISA assays were performed following the manufacturer’s instructions for each kit (Mouse IL-6 ELISA Kit, ABclonal, Wuhan, China; Mouse TNF-alpha ELISA Kit, ABclonal; Mouse Kynurenine ELISA Kit, Finetest, Wuhan, China; Human Kynurenine ELISA Kit, Finetest; Trp, ELK Biotechnology, Wuhan, China). The procedure included sample loading, incubation, and washing steps. Optical density values were measured using a microplate reader at the specified wavelength, and standard curves were generated from known concentrations to quantify the sample concentrations.

### Bacterial plating assay

Systemic bacteremia was quantified through standardized culture techniques. Peripheral blood samples were collected aseptically and diluted 100-fold in sterile PBS. Aliquots (100 μL) were evenly spread onto LB agar plates using a sterile L-shaped spreader. Culture plates were incubated inverted at 37 °C for 16 h to allow bacterial growth. Bacterial burden was subsequently determined through manual quantification of colony-forming units (CFUs), with counts normalized to original blood volume.

### Chromatin immunoprecipitation (ChIP) assay

The ChIP assay was performed using a Chromatin Immunoprecipitation Kit (BersinBio, Guangzhou, China) following the manufacturer’s instructions. Thymocytes were isolated from sham and CLP mice 24 h post-surgery for chromatin preparation. An anti-aryl hydrocarbon receptor (AhR) antibody (Enzo Life Sciences, Farmingdale, NY, USA) was used for immunoprecipitation, RT-qPCR was conducted using primers specifically designed to amplify the putative xenobiotic response element (XRE) binding sites within the *Pla2g4a* promoter region. The primer sequences used are provided in Table S2.

### Statistical analysis

All the statistical analyses and plots were conducted using GraphPad Prism v9 software (San Diego, CA, USA). Comparisons between groups were performed using either the Mann-Whitney U test or Student’s *t*-test. For multiple group comparisons, the Kruskal-Wallis test followed by Dunn’s test, or One-way ANOVA followed by Tukey’s test, was used as appropriate. Simple linear regression was used to analyze relationships between variables. Each experiment was repeated independently three times to ensure reproducibility. Biological replicates times are indicated in the figure legends. Bars represent the means ± SEM. All *P*-values were two-tailed, and values <0.05 were considered statistically significant.

For detailed information on materials, see the Table S3.

## Supplementary information

Full and uncropped western blots

Supplementary figures and tables

## Data Availability

The paper is present in the main text or the supplementary materials. Additional data related to this paper may be requested from the authors.
