# Supplementary figures and images for: Ferroptosis mediated by the IDO1/Kyn/AhR pathway triggers acute thymic involution in sepsis
Source: Cell Death Dis. 2025 Jul 25;16(1):562. doi: 10.1038/s41419-025-07882-9 (PMC12297531; doi:10.1038/s41419-025-07882-9)

Figure 6A

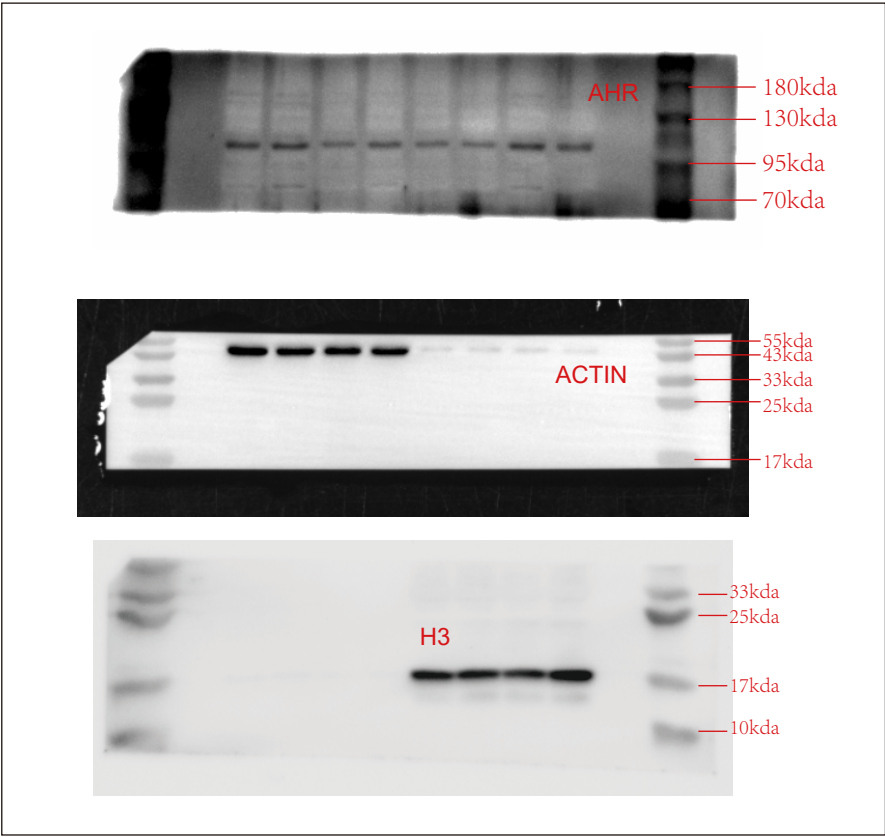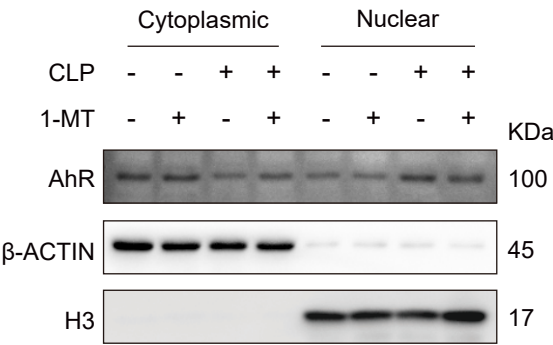

Figure 6B

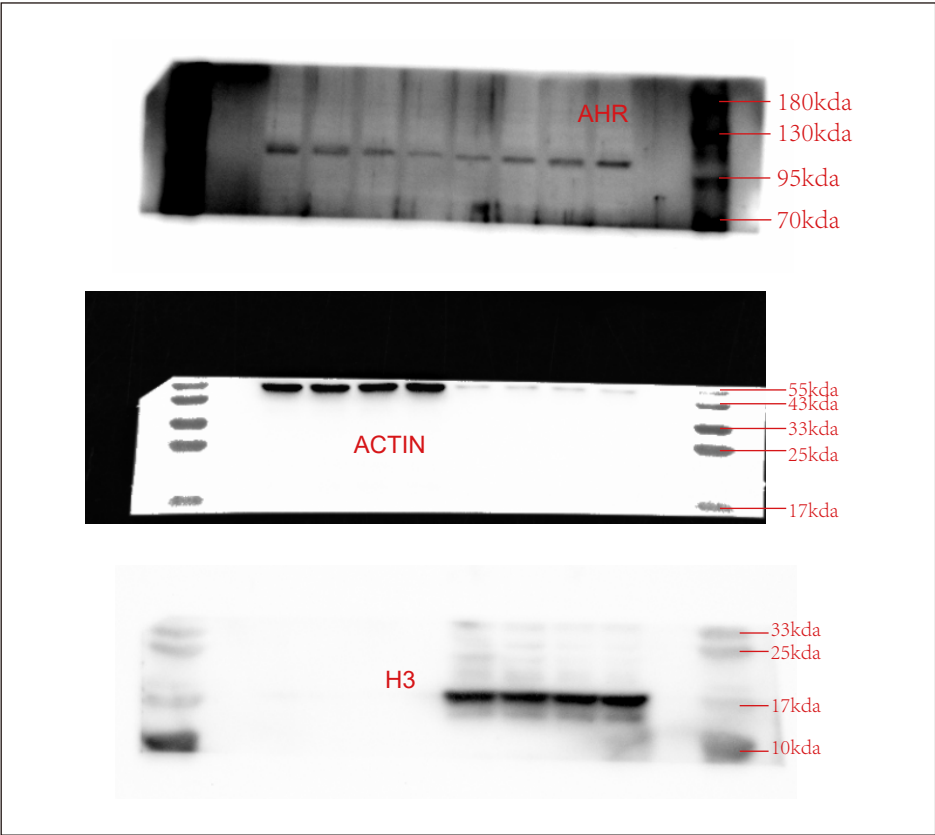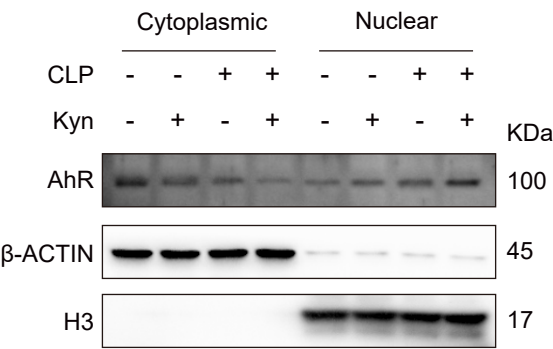

Figure S12A

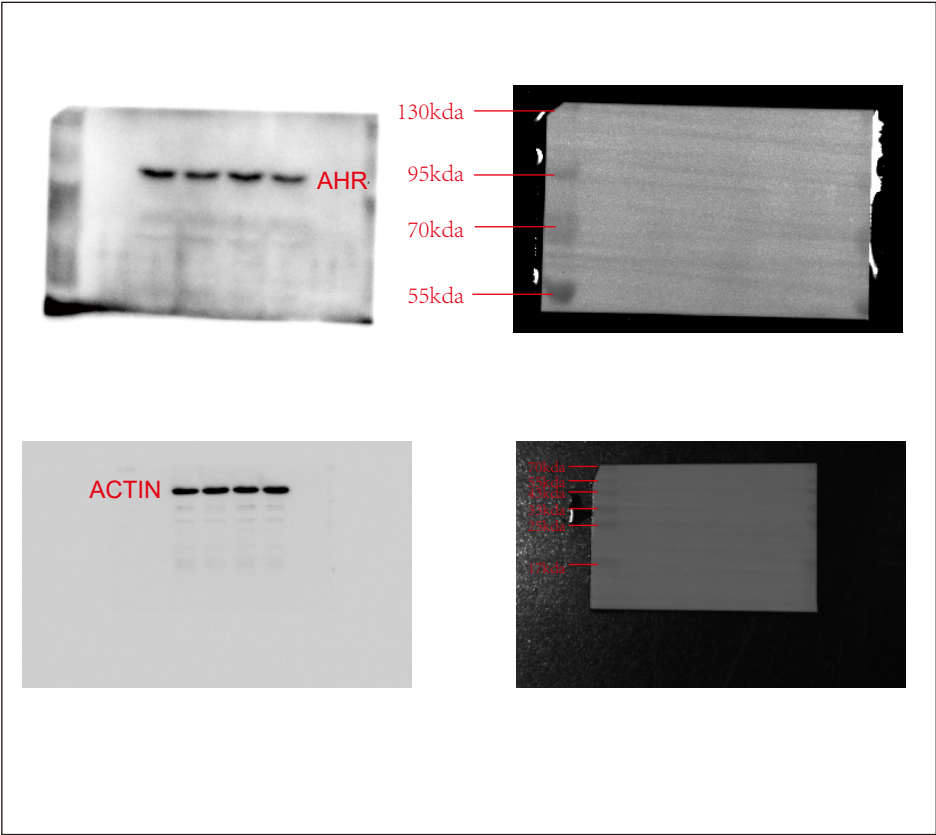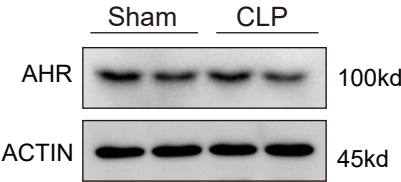

Figure S12C

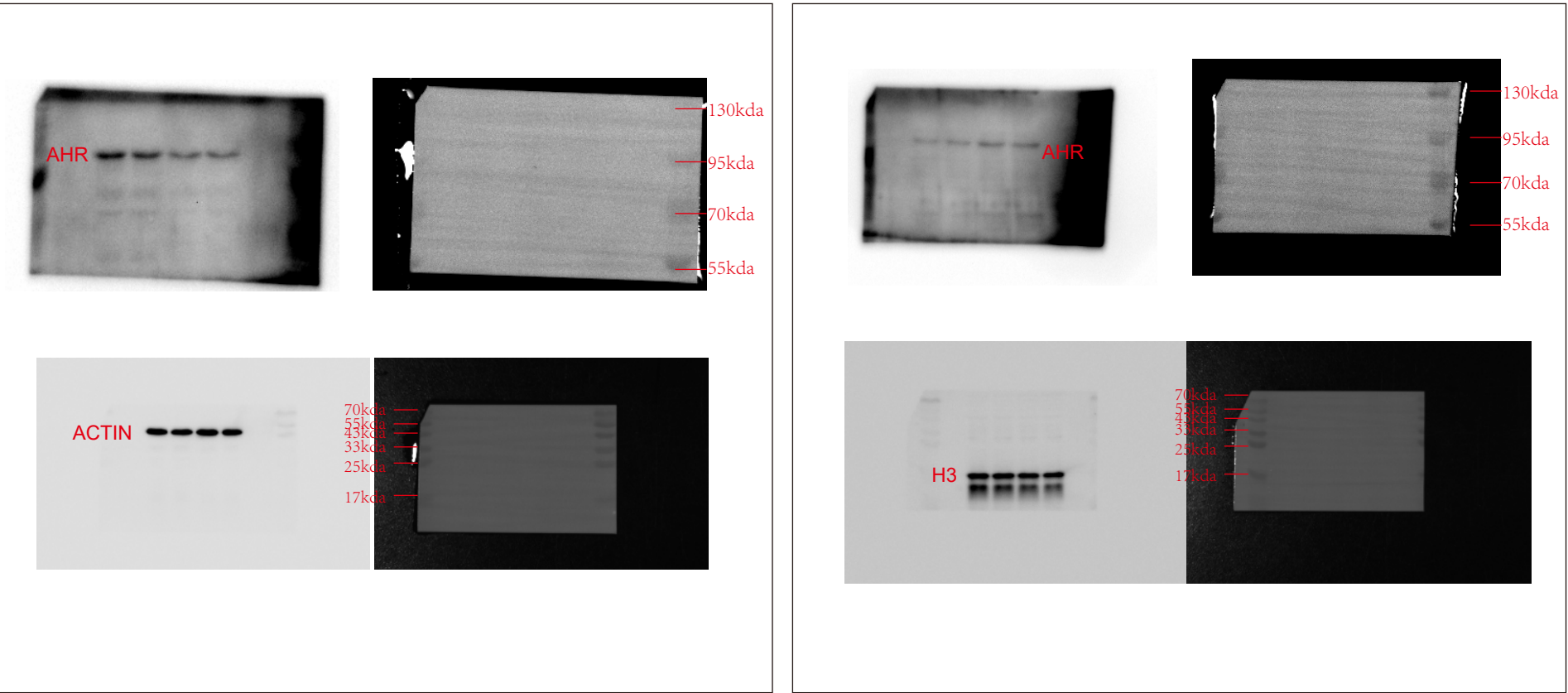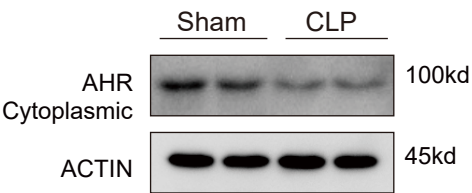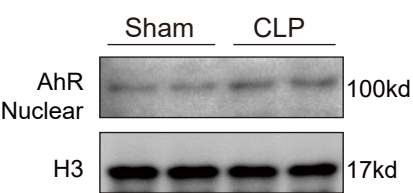

Supplement: Supplementary file 1 — Full and uncropped western blots [file 41419_2025_7882_MOESM1_ESM.pdf]
