# Supplementary material for: Ferroptosis mediated by the IDO1/Kyn/AhR pathway triggers acute thymic involution in sepsis
Source: Cell Death Dis. 2025 Jul 25;16(1):562. doi: 10.1038/s41419-025-07882-9 (PMC12297531; doi:10.1038/s41419-025-07882-9)
Supplement: Supplementary file 2 — Supplementary figures and tables [file 41419_2025_7882_MOESM2_ESM.pdf]

This file includes:

Figs. S1 to S15

Tables S1 to S3

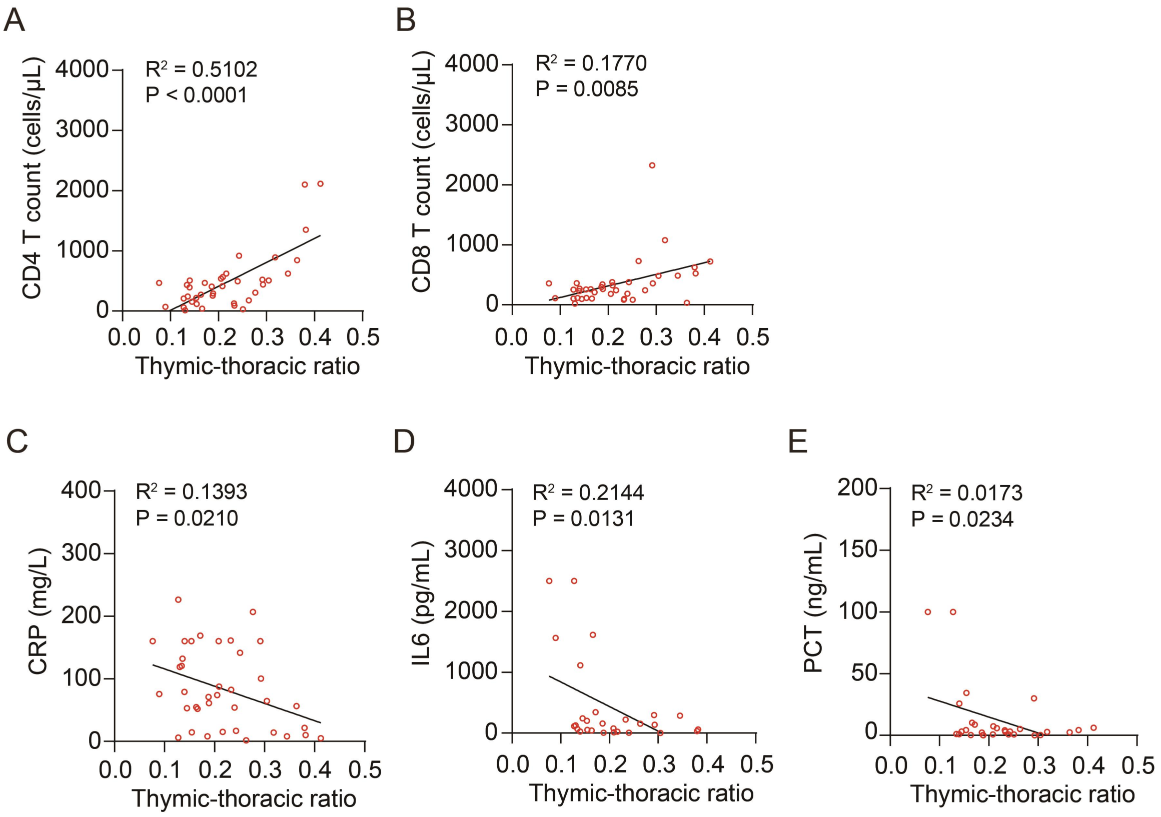

Fig. S1. Correlation analysis of thymic-thoracic ratio with peripheral blood T cells and inflammatory markers in pediatric sepsis patients.

(**A-B**) Corelation between the thymus-to-thoracic ratio and CD4 T cell count (**A**) (n=38), and CD8 T cell count (**B**) (n=38). (**C-E**) Corelation between the thymus-to-thoracic ratio and inflammatory markers, including CRP (**C**) (n=38), IL-6 (**D**) (n=28), and PCT (**E**) (n=30).

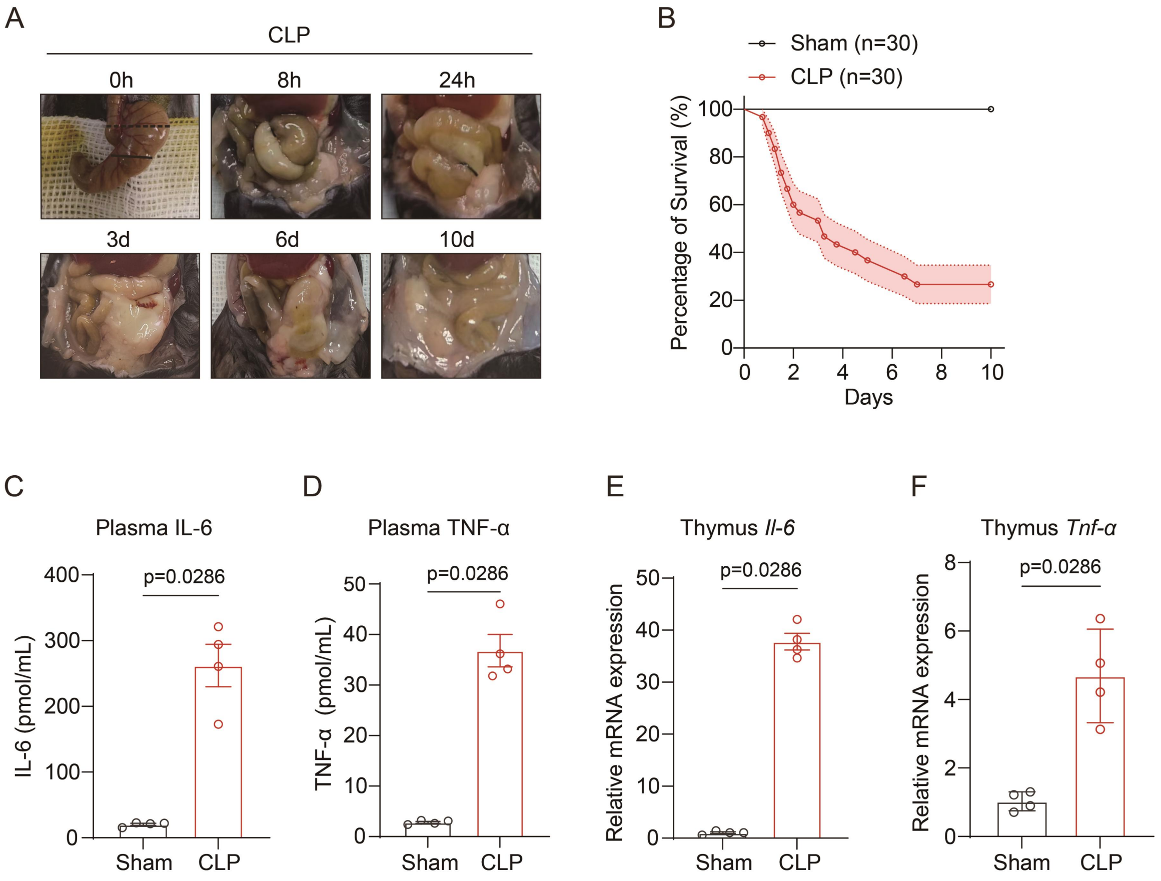

Fig. S2. Construction of the sepsis mouse model.

(**A**) Representative images showing the abdominal condition of mice at 0h, 8h, 24h, 3 days, 6 days, and 10 days post-CLP surgery. (**B**) Survival curve of sham and CLP-treated mice (n=30). (**C-D**) Plasma levels of IL-6 (**C**) and TNF-α (**D**) in sham and CLP mice on day 1 post-CLP (n=4). (**E-F**) Relative mRNA expression levels of *Il-6* (**E**) and *Tnf-α* (**F**) in thymocytes from sham and CLP mice on day 1 post-CLP (n=4). Bars represent the means ± SEM.

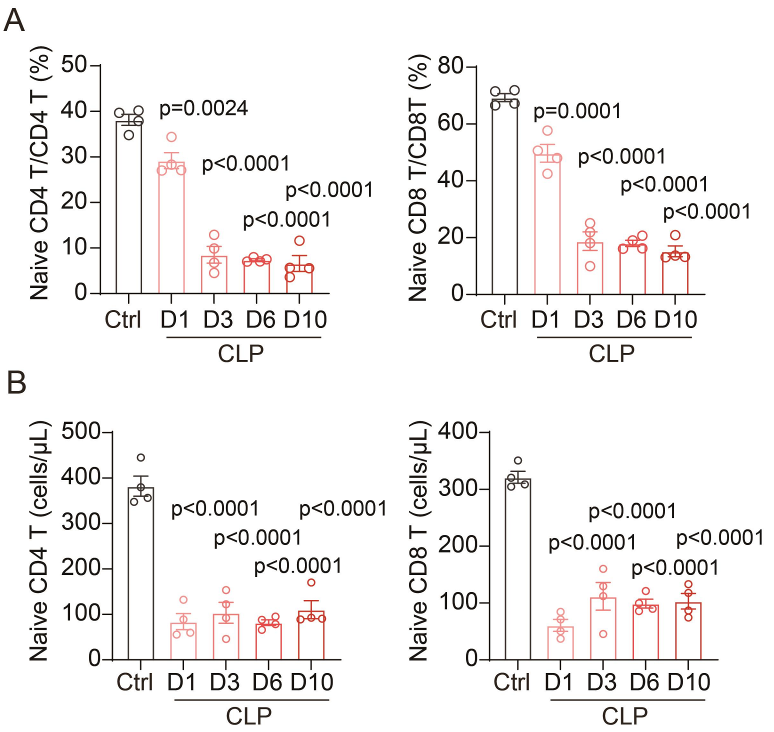

Fig. S3. Proportion and absolute count of T cells in polymicrobial sepsis mouse model.

(**A**) Proportion of naive $CD4^+$ T cells and $CD8^+$T cells in peripheral blood over time (n=4). (**B**) Absolute counts of naive $CD4^+$ and $CD8^+$ T cells in peripheral blood over time (n=4). Bars represent the means ± SEM.

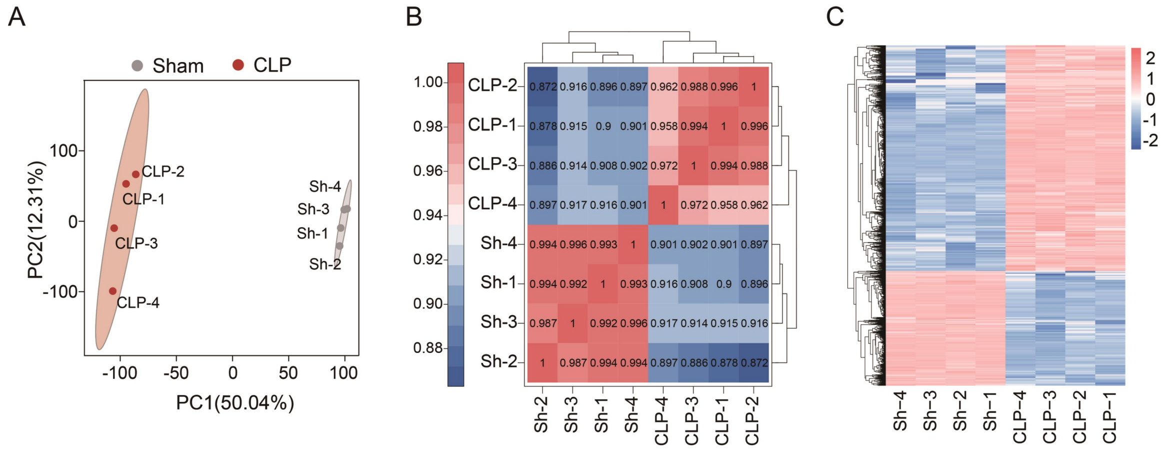

Fig. S4. Transcriptomic analysis in the CLP model.

(**A**) Principal component analysis (PCA) of RNA-seq data comparing thymocytes from sham and CLP-treated mice. (**B**) Correlation heatmap showing hierarchical clustering of RNA-seq gene expression profiles from sham and CLP-treated thymocytes. (**C**) Heatmap of differentially expressed genes in thymocytes from CLP versus sham mice.

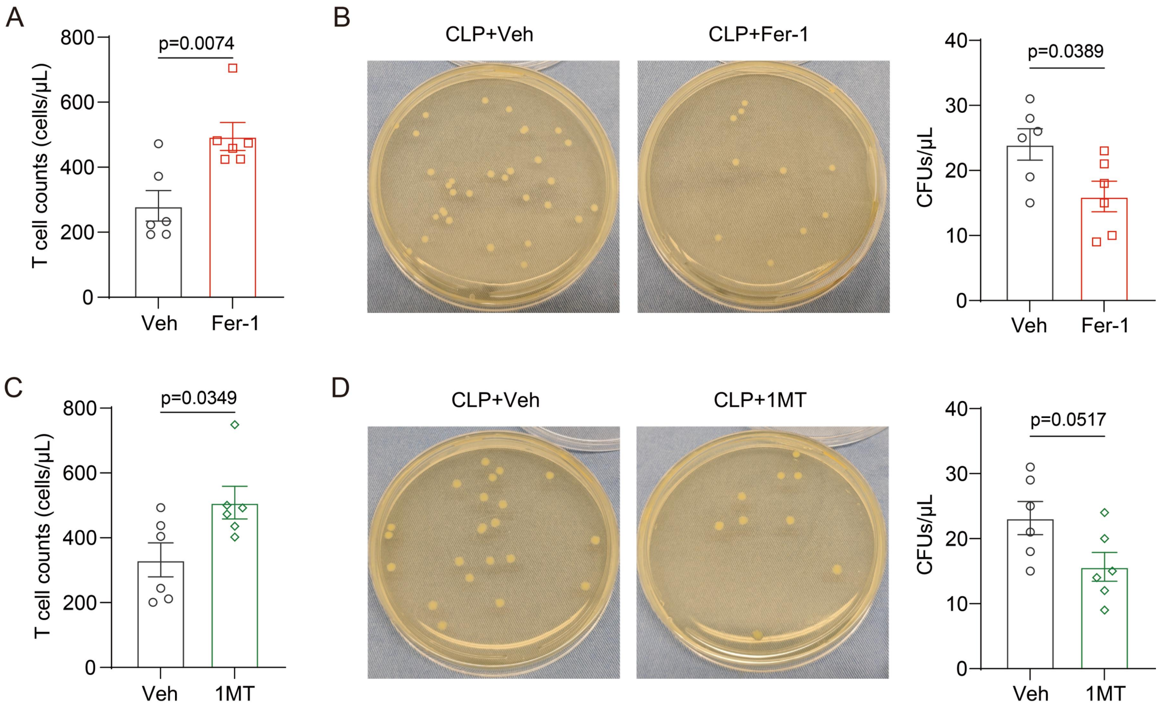

Fig. S5. Ferroptosis inhibition and IDO1 blockade restore T-cell counts and enhance bacterial clearance in sepsis mice.

(**A**) Peripheral blood T-cell counts in CLP mice treated with Fer-1 or vehicle (n=6). (**B**) Bacterial colony-forming unit (CFU) assay of blood samples from CLP mice treated with Fer-1 or vehicle (n=6). (**C**) Peripheral blood T-cell counts in CLP mice treated with 1-MT or vehicle (n=6). (**D**) Bacterial CFU assay of blood samples from CLP mice treated with 1-MT or vehicle (n=6). Bars represent the means ± SEM.

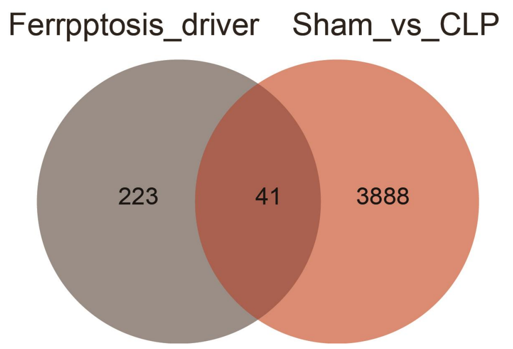

Fig. S6. Analysis of ferroptosis driver genes in thymocytes.

Venn diagram showing the overlap between ferroptosis driver genes and differentially expressed genes identified through RNA-seq analysis in sham versus CLP thymocytes.

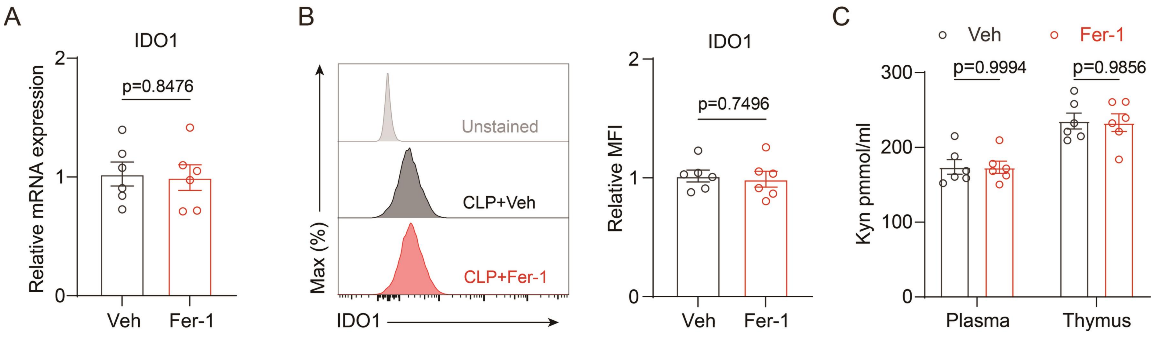

Fig. S7. Fer-1 does not affect IDO1 expression or kynurenine levels in septic mice.

(**A**) RT-qPCR analysis of *Ido1* mRNA expression in the thymus of CLP mice treated with Fer-1 or vehicle (n=6). (**B**) Flow cytometric analysis of IDO1 protein levels in thymocytes from CLP mice treated with Fer-1 or vehicle (n=6). (**C**) ELISA quantification of kynurenine (Kyn) concentrations in plasma and thymus of CLP mice treated with Fer-1 or vehicle (n=6). Bars represent the means ± SEM.

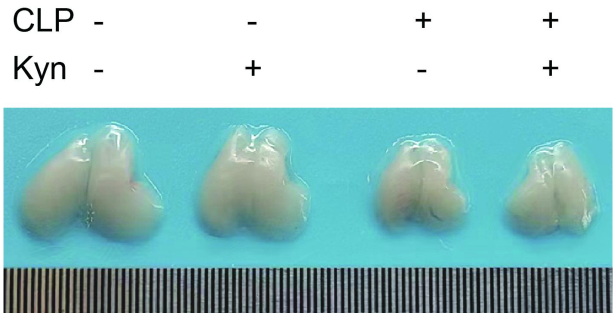

Fig. S8. Representative image of thymus after Kyn administration.

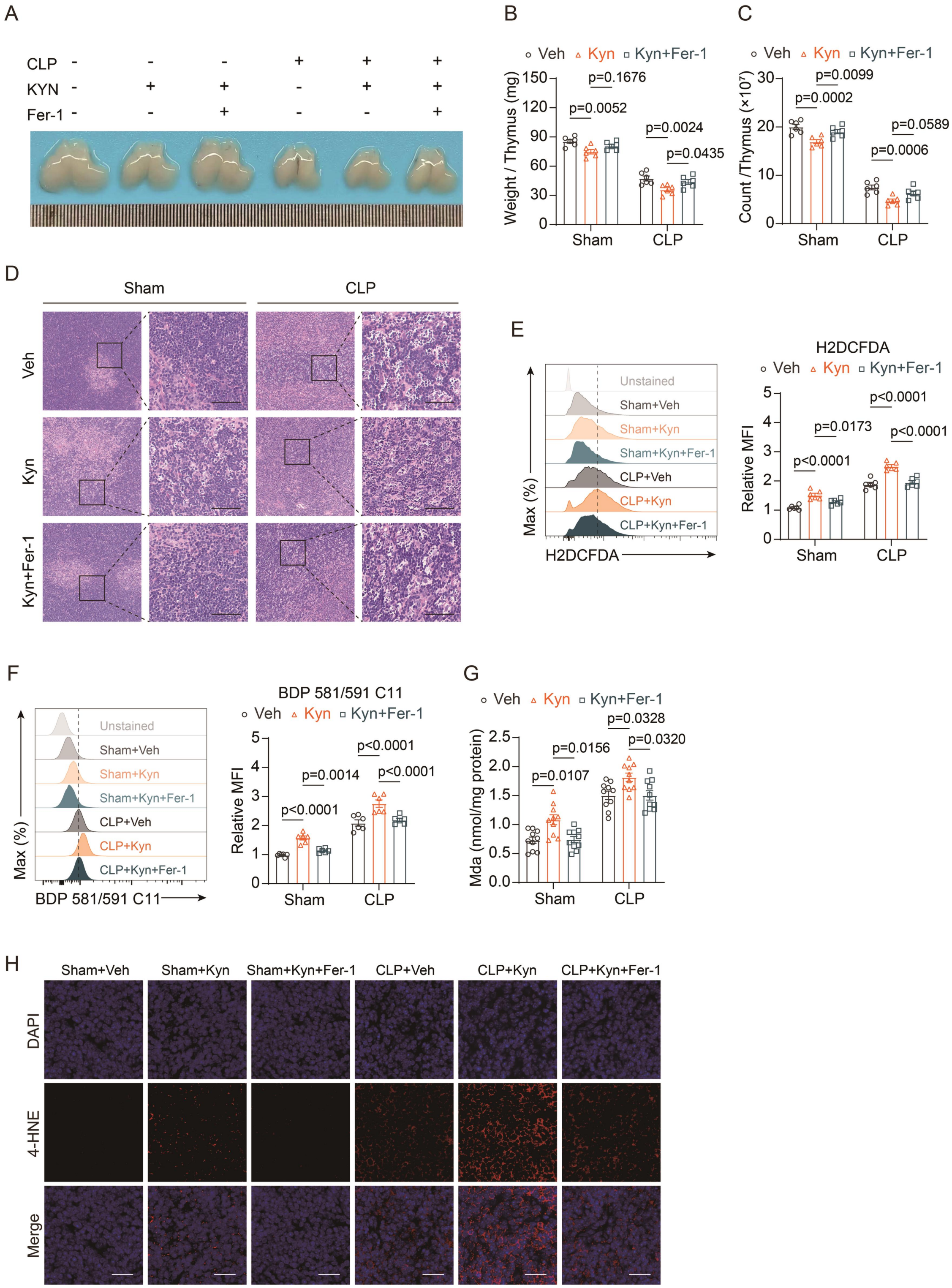

Fig. S9. Fer-1 attenuates Kyn-induced ATI and suppresses lipid peroxidation in thymocytes. (**A-C**) Representative images of thymus morphology (**A**), and quantification of thymus weight (**B**) and thymocyte counts (**C**) in sham and CLP mice after treatment with Kyn and ferroptosis

inhibitor Fer-1, compared to Kyn or vehicle alone (n=6). (**D**) Representative HE staining of thymocytes from sham and CLP mice treated with Kyn and Fer-1, compared to Kyn or vehicle alone (scale bar=50 μm). (**E-F**) Flow cytometry analysis of ROS production (**E**) and lipid peroxidation (**F**) in thymocytes from sham and CLP mice treated with Kyn and Fer-1, compared to Kyn or vehicle alone (n=6). (**G**) MDA levels in thymic tissue from sham and CLP mice treated with Kyn and Fer-1, compared to Kyn or vehicle alone (n=10). (**H**) Immunofluorescence staining of 4-HNE in thymic sections from sham and CLP mice treated with Kyn and Fer-1, compared to Kyn or vehicle alone (scale bar=25 μm).

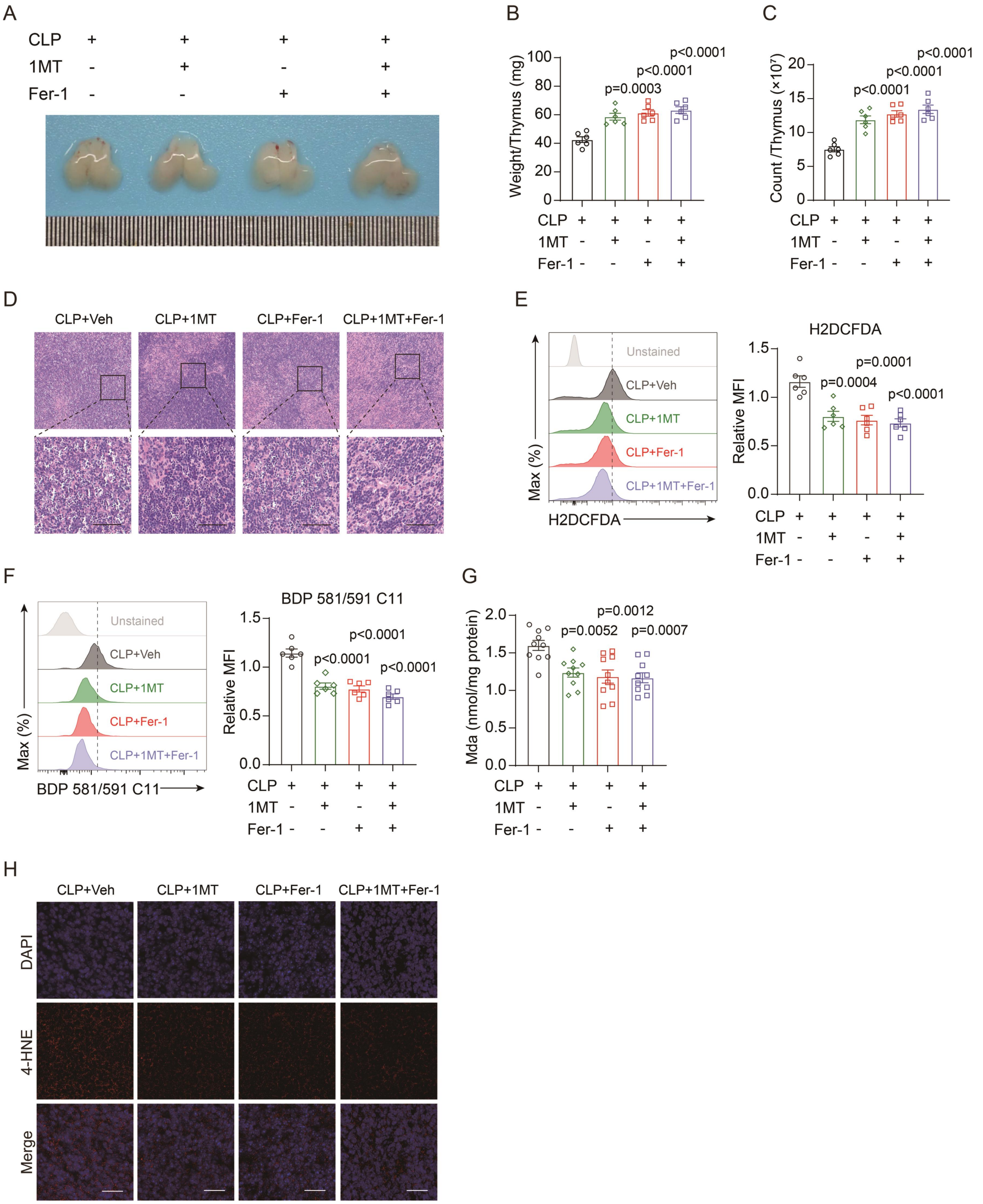

Fig. S10. Dual inhibition of IDO1 and ferroptosis mitigates sepsis-induced thymic atrophy but lacks synergy.

(**A-C**) Representative images of thymus morphology (**A**), and quantification of thymus weight (**B**) and thymocyte counts (**C**) in CLP mice after treatment with vehicle, 1-MT, Fer-1, or their

combination (n=6). (**D**) Representative HE staining of thymocytes from CLP mice treated with vehicle, 1-MT, Fer-1, or their combination (scale bar=50 μm). (**E-F**) Flow cytometry analysis of ROS production (**E**) and lipid peroxidation (**F**) in thymocytes from CLP mice treated with vehicle, 1-MT, Fer-1, or their combination (n=6). (**G**) MDA levels in thymic tissue from CLP mice treated with with vehicle, 1-MT, Fer-1, or their combination (n=10). (**H**) Immunofluorescence staining of 4-HNE in thymic sections from CLP mice treated with vehicle, 1-MT, Fer-1, or their combination (scale bar=25 μm). Bars represent the means ± SEM.

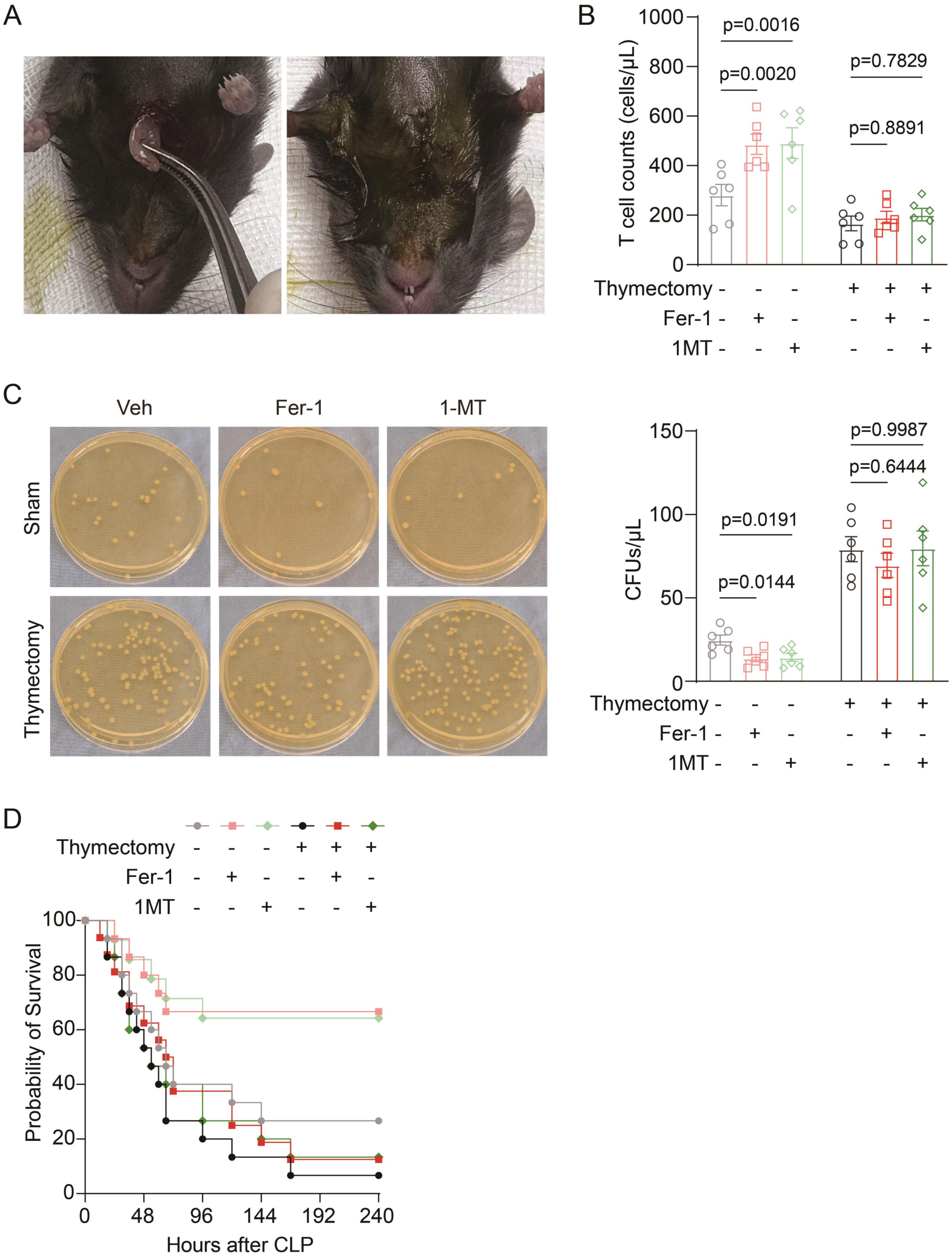

Fig. S11. Inhibition of ferroptosis improves survival in septic mice by targeting the thymus.

(A) Schematic illustration of thymectomy in mice. (B) T-cell counts in thymectomized CLP mice treated with Fer-1 or 1-MT (n = 6). (C) Bacterial burden in peripheral blood assessed by CFU assay following treatment with Fer-1 or 1-MT in thymectomized CLP mice (n = 6). (D) Kaplan–Meier survival curves of thymectomized CLP mice receiving Fer-1 or 1-MT (n = 15). Data are presented as means ± SEM.

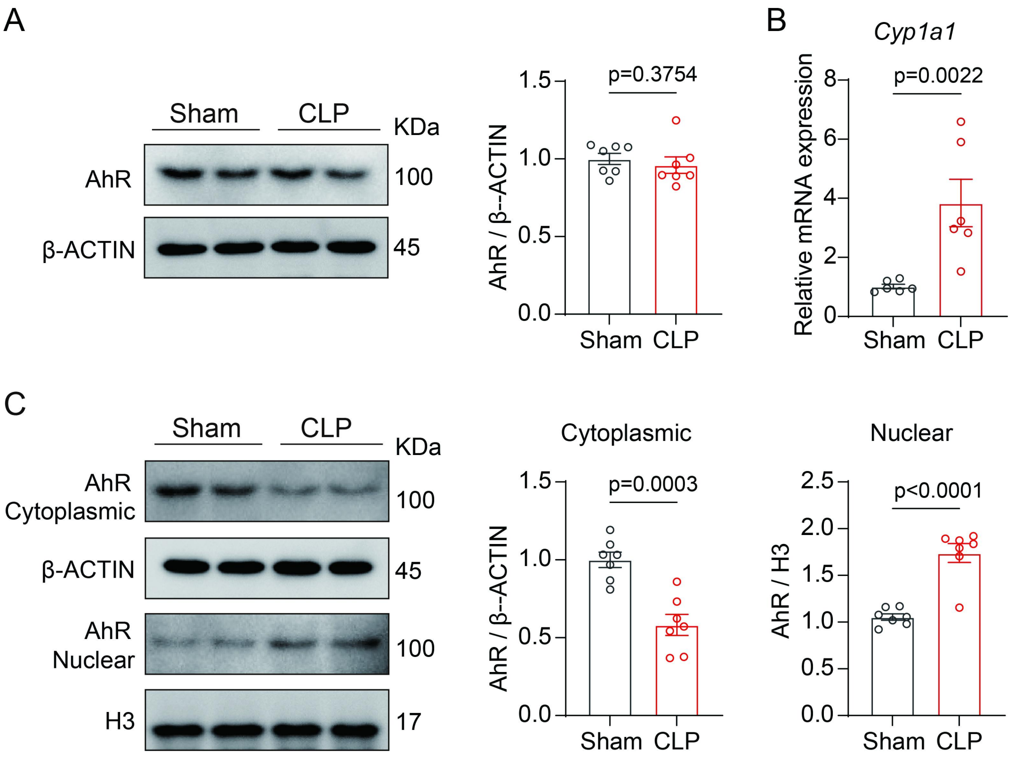

Fig. S12. Increased nuclear translocation of AhR in thymocytes triggered by sepsis.

(**A**) Western blot (WB) analysis showing the expression of AhR in thymocytes from sham and CLP mice (n=8). (**B**) Relative *Cyp1a1* mRNA expression in thymocytes from sham and CLP mice (n=6). (**C**) Western blot analysis showing the relative AhR expression in the nucleus and cytoplasm of thymocytes from sham and CLP mice (n=7). Bars represent the means ± SEM.

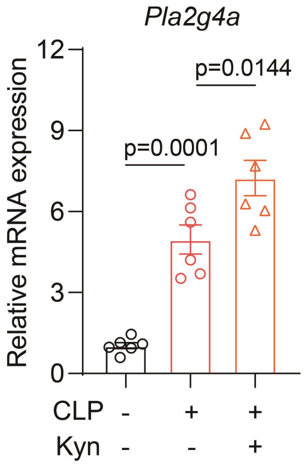

Fig. S13. Relative *Pla2g4a* mRNA expression in thymocytes (n=6). Bars represent the means ± SEM.

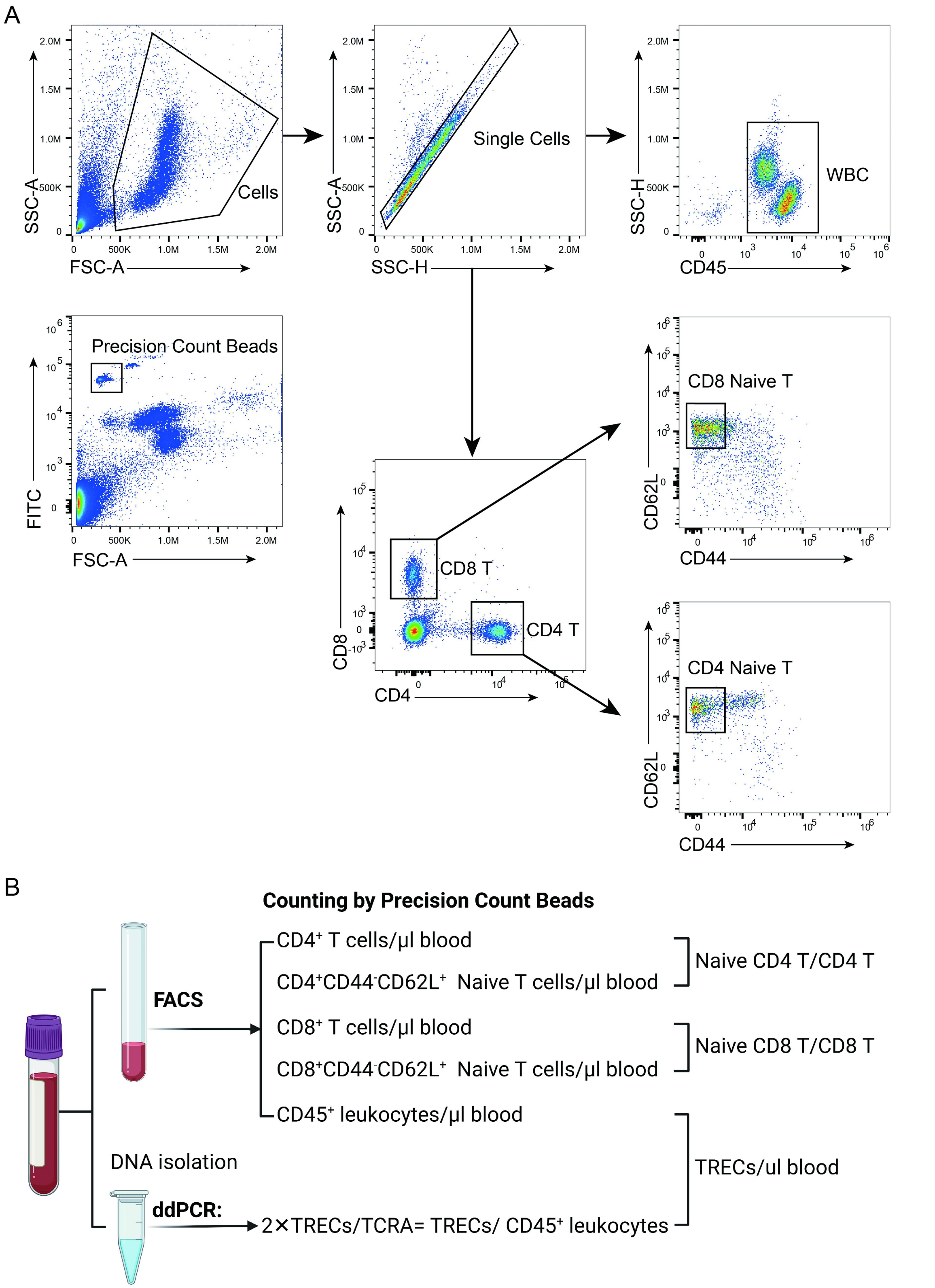

Fig. S14. Flow cytometry gating strategy and TRECs analysis procedure.

(**A**) Flow cytometry gating strategy for identifying white blood cells (WBC), CD4$^+$ and CD8$^+$ T cells, and naive CD4$^+$ and CD8$^+$ T cell populations in peripheral blood, using precision count beads for absolute cell counting. (**B**) Schematic of the TRECs (T-cell receptor excision circles) analysis procedure. Flow cytometry (FACS) is used to determine the absolute counts of CD4$^+$ T cells, CD8$^+$ T cells, and naive T cell subsets. DNA is isolated for digital droplet PCR (ddPCR) to quantify TRECs per microliter of blood.

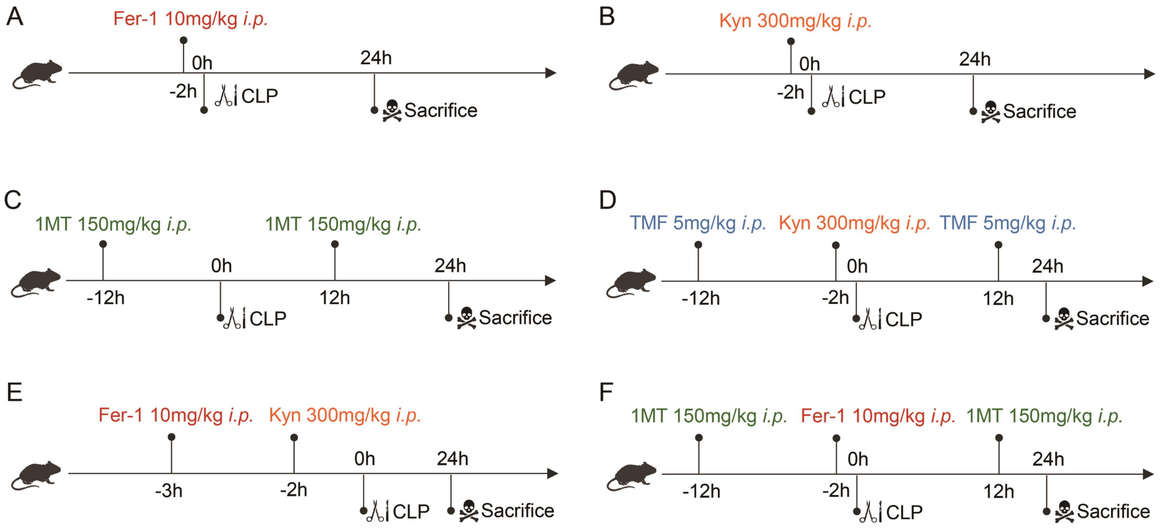

Fig. S15. Dosing regimen for mice in sepsis model experiments.

(**A**) Ferrostatin-1 (Fer-1) was administered at 10 mg/kg intraperitoneally (*i.p.*) 2 hours before CLP, and mice were sacrificed 24 hours post-CLP. (**B**) Kynurenine (Kyn) was administered at 300 mg/kg *i.p.* 2 hours before CLP. (**C**) 1-Methyltryptophan (1-MT) was administered at 150 mg/kg *i.p.* 12 hours before and 12 hours after CLP. (**D**) TMF (5 mg/kg *i.p.*) was administered 12 hours before and 12 hours after CLP, while Kyn (300 mg/kg *i.p.*) was administered 2 hours before CLP. (**E**) Fer-1 was administered at 10 mg/kg intraperitoneally (*i.p.*) 3 hours before CLP, while Kyn (300 mg/kg *i.p.*) was administered 2 hours before CLP. (**F**) 1-MT was administered at 150 mg/kg *i.p.* 12 hours before and 12 hours after CLP, while Fer-1 was administered at 10 mg/kg intraperitoneally (*i.p.*) 2 hours before CLP.

Table S1. Characteristics of sepsis and healthy control group

Characteristics	Sepsis	Control	*P* value
male/female	22/16	18/10	0.599
Age, years, M (IQR)	5.2 (1.8, 8.3)	6.4 (3.5, 10.9)	0.112
Site of initial infection			
Respiratory tract	28	NA	NA
Digestive tract	7	NA	NA
Intracranial	1	NA	NA
Urinary_tract	2	NA	NA
Organ support			
Mechanical ventilation	24	NA	NA
Hemopurification	15	NA	NA
ECMO	3	NA	NA
Pathogen			
Gram-negative bacteria	7	NA	NA
Gram-positive bacteria	6	NA	NA
Virus	4	NA	NA
Others	21	NA	NA
p-SOFA, M (IQR)	5.5 (3, 8)	NA	NA
CRP, mg/L, M (IQR)	72.3 (16.2, 146.2)	NA	NA
PCT[#], ng/mL, M (IQR)	3.2 (0.8, 7.7)	NA	NA
IL-6[§], pg/mL, M (IQR)	132.4 (34.0, 293.4)	NA	NA
WBC, $\times 10^3$ ul^{-1}, M (IQR)	8.9 (4.0, 20.8)	NA	NA
Total T cell count, ul^{-1}, M (IQR)	722.6 (355.4, 920.1)	NA	NA
CD4 T cell count, ul^{-1}, M (IQR)	409.3 (170.2, 542.9)	NA	NA
CD8 T cell count, ul^{-1}, M (IQR)	256.7 (112.4, 376.4)	NA	NA
Died/survived	10/28	NA	NA

Abbreviations: IQR, inter-quartile range; ECMO, extracorporeal membrane oxygenation; p-SOFA, pediatric sequential organ failure assessment score; CRP, C-reactive protein; PCT, procalcitonin; IL-6, Interleukin-6; WBC, white blood cell. [#] There are 8 missing values for PCT. [§] There are 10 missing values for IL-6.

Table S2. Primer sequence

Gene	Organism	Sequence	
TREC	*Homo sapiens*	Forward	5′-CACATCCCTTTCAACCATGCT-3′
		Reverse	5′-TGCAGGTGCCTATGCATCA-3′
		Probe	5′-ACACCTCTGGTTTTTGTAAAGGTGCCCACT-3′
TCRA	*Homo sapiens*	Forward	5′-TGTCCTAACCCTGATCCTCTT-3′
		Reverse	5′-GGATTTAGAGTCTCTCAGCTGGTACAC-3′
		Probe	5′-TCCCACAGATATCCAGAACCCTGACCC-3′
Trec	*Mus musculus*	Forward	5′-CCAAGCTGACGGCAGGTTT-3′
		Reverse	5′-AGCATGGCAAGCAGCACC-3′
		Probe	5′-TGCTGTGTGCCCTGCCCTGCC-3′
Tcra	*Mus musculus*	Forward	5′-TGACTCCCAAATCAATGTGCCG-3′
		Reverse	5′-GCAGGTGAAGCTTGTCTGGTTG-3′
		Probe	5′-AAACTGTGCTGGACATGAAAGCTATGGAT-3′
Il-6	*Mus musculus*	Forward	5′-TACCACTTCACAAGTCGGAGGC-3′
		Reverse	5′-CTGCAAGTGCATCATCGTTGTTC-3′
Tnf-α	*Mus musculus*	Forward	5′-GGTGCCTATGTCTCAGCCTCTT-3′
		Reverse	5′-GCCATAGAACTGATGAGAGGGAG-3′
Ptgs2	*Mus musculus*	Forward	5′-GCGACATACTCAAGCAGGAGCA-3′
		Reverse	5′-AGTGGTAACCGCTCAGGTGTTG-3′
Acsl3	*Mus musculus*	Forward	5′-GCGAGAAGGATTCCAAGACTGG-3′
		Reverse	5′-GAAGAGTAGCCGATTCGGCATC-3′
Pla2g4	*Mus musculus*	Forward	5′-GATGAGGCTCAAGGACCCAAAG-3′
		Reverse	5′-GAATAAAGCCGAGTCGCTCACC-3′
Alox5	*Mus musculus*	Forward	5′-GACACTTGGTGGCTGAGGTCTT-3′
		Reverse	5′-TCTCTGAGATCAGGTCGCTCCT-3′
Alox15	*Mus musculus*	Forward	5′-GACACTTGGTGGCTGAGGTCTT-3′
		Reverse	5′-TCTCTGAGATCAGGTCGCTCCT-3′
Ido1	*Mus musculus*	Forward	5′-GCAGACTGTGTCCTGGCAAACT-3′
		Reverse	5′-AGAGACGAGGAAGAAGCCCTTG-3′
XRE1	*Mus musculus*	Forward	5′-CCCAGGCGGGAGTGCAATGG-3′
		Reverse	5′-ATTAGCCGGGTGTGGTGGCG-3′
XRE2	*Mus musculus*	Forward	5′-TCCTGACCTCGTGATCCGCC-3′
		Reverse	5′-TTTTTCGGGCTGGGCACGGT-3′

Table S3. Key resources table

Reagent	Source	Country	Identifier
Precision Count Beads™	BioLegend	USA	424902
FITC anti-mouse CD45.2 Antibody	BioLegend	USA	109805
PE/Cyanine7 anti-mouse CD4 Antibody	BioLegend	USA	100422
PE anti-mouse CD8a Antibody	BioLegend	USA	162304
PE/Cyanine5 anti-mouse/human CD44	BioLegend	USA	103010
FITC anti-mouse CD62L Antibody	BioLegend	USA	104405
Alexa Fluor® 647 anti-IDO1 Antibody	BioLegend	USA	654003
PI dye	BD Biosciences	USA	556547
TIANamp Blood DNA Kit	Tiangen Biotech	China	DP348
RNAiso Plus	Takara	Japan	9108
PrimeScript RT Master Mix kit	Takara	Japan	RR036A
Iron Assay Kit	Dojindo	Japan	I291
H2DCFDA	MCE	USA	HY-D0940
BDP 581/591 C11	Dojindo	Japan	L267
MDA assay kit	Nanjing Jiancheng	China	A00341
anti-4 hydroxynonenal antibody	Abcam	UK	ab46545
Cy3 conjugated Goat Anti-Rabbit IgG (H+L)	Servicebio	China	GB21303
Antifade Mounting Medium with DAPI	Beyotime	China	P0131
Ferrostatin-1	MCE	USA	HY100579
Kynurenine	MCE	USA	HY104026
6,2',4'-Trimethoxyflavone	MCE	USA	HY-103220
1-Methyltryptophan	Sigma	USA	447439
RIPA Lysis Buffer	Beyotime	China	P0013K
Halt Protease Inhibitor Cocktail	Thermo Fisher Scientific	USA	87785
Nuclear and Cytoplasmic Protein Extraction	Beyotime	China	P0027
SDS-PAGE Gel Quick Preparation Kit	Beyotime	China	P0012AC
Protein Free Rapid Blocking Buffer	Epizyme Biomedical	China	PS108P
AhR antibody	Enzo Life Sciences	USA	BML-SA210-
β-Actin Antibody	CST	USA	4967
Histone H3 Antibody	CST	USA	9715
Goat Anti-Rabbit IgG-HRP	Abmart	China	M21002
Mouse IL-6 ELISA Kit	ABclonal	China	RK00008
Mouse TNF-alpha ELISA Kit	ABclonal	China	RK00027
Mouse Kynurenine ELISA Kit	Finetest	China	EM1862
Human Kynurenine ELISA Kit	Finetest	China	EH4214

Tryptophan ELISA Kit	ELK Biotechnology	China	ELK8262
LB agar medium	BKMAMLAB	China	110704002
Chromatin Immunoprecipitation Kit	BersinBio	China	Bes5001
